# What works to reduce loneliness: a rapid systematic review of 101 interventions

**DOI:** 10.1057/s41271-025-00561-1

**Published:** 2025-03-06

**Authors:** Joanna M. Blodgett, Katie Tiley, Frances Harkness, Margherita Musella

**Affiliations:** 1 Kohlrabi, Manchester, SK4 3HJ UK; 2 Division of Surgery & Interventional Science, Institute of Sport Exercise & Health, University College London, 170 Tottenham Court Road, London, W1T 7HA UK; 3 Centre for Mathematical Modelling of Infectious Diseases, London School of Hygiene & Tropical Medicine, London, WC1E 7HT UK; 4 Department of Infectious Disease Epidemiology, Faculty of Epidemiology and Population Health, London School of Hygiene & Tropical Medicine, London, WC1E 7HT UK; 5 What Works Centre for Wellbeing, London, SW1H 9EA UK

**Keywords:** Evaluation, Intervention, Loneliness, Meta-analysis, Pre-post, Rapid review

## Abstract

This review evaluates the effectiveness of interventions aimed at reducing loneliness across all age groups. Studies were eligible if alleviation of loneliness was one of ≤ 3 aims, and loneliness was assessed pre- and post-intervention using a validated quantitative measure. Random-effects meta-analyses of standardised mean differences (SMD) assessed pre–post-intervention differences in loneliness, including comparing control and intervention groups. A comprehensive search of peer-reviewed and grey literature sources identified 95 studies covering 101 interventions. Psychological interventions had the largest SMD effect size (*n* = 23: − 0.79 [95%CI: − 1.19, − 0.38]), followed by social interaction-based interventions (*n* = 23; − 0.50 [− 0.78, − 0.17]), social support-based interventions (*n* = 46; − 0.34 [− 0.45, − 0.22]), and finally interventions involving multiple themes (*n* = 9). Findings highlight the effectiveness of psychological and social interaction strategies in reducing loneliness. Future research should address gaps related to intervention types and populations, emphasising randomised controlled designs. Policymakers and practitioners can use these insights to prioritise interventions fostering social connections and psychological support across diverse settings.

**Supplementary Information:**

The online version contains supplementary material available at 10.1057/s41271-025-00561-1.

## Key Messages

- A wide variety of interventions can be successful at improving loneliness in adults of all ages.
- Interventions focussing on psychological therapy and structured social activities are the most effective at reducing loneliness across all age groups, although interventions designed to address loneliness through targeted emotional and social support are also effective.
- Future interventions should prioritise randomized controlled designs to better understand which approaches work best for specific populations, ensuring effective policies and resource allocation to tackle loneliness on a broader scale.

**Supplementary Information:**

The online version contains supplementary material available at 10.1057/s41271-025-00561-1.

## Introduction

Over two thirds of individuals will report being lonely at some stage in their life, with higher levels of loneliness in early or later life [1]. Definitions of loneliness are widely based on the cognitive model which defines the concept as “a subjective, unwelcome feeling of lack or loss of companionship… a mismatch between the quantity and quality of social relationships that we have, and those that we want”. [2, 3]. The impact of loneliness on the body can be significant, leading to greater risk of depression [4], pain and fatigue [5], and overall poorer health. According to the cognitive model of loneliness, the phenomenon is inherently psychological and distinct from social isolation [6], as it is influenced by perceived quality of social interactions rather than the quantity of interactions with others. Interventions aiming to alleviate loneliness can be delivered directly (for example, increase social opportunities such as dance classes or group activities [7]) or indirectly (e.g. educate and develop skills for individuals to combat loneliness such as cognitive behavioural therapy [8, 9]).

Historically, research in this field has focussed on older populations in community settings and care homes [6, 10–13] or children and adolescents [10, 11, 14], despite individuals of all ages experiencing loneliness, as demonstrated by the universal response to the social isolation induced by the COVID-19 pandemic [15–19]. In recent years, there is an increasing number of loneliness interventions aimed at specific populations including migrant populations [20] or those with clinical psychological diagnoses [21] as well as those utilising diverse and novel delivery mechanisms [22]. However, a common theme across previous reviews is the call for more targeted and higher quality approaches to the delivery and evaluation of loneliness interventions [1, 23].

There is now a larger and richer body of evidence exploring loneliness interventions across all age groups. This rapid systematic review and meta-analyses aim to address this gap and provide insight into effective loneliness interventions across the entire life-course and across any intervention theme. This review aims to inform research, policy, and practice by answering the research question: what is the effectiveness of interventions aimed at alleviating loneliness in people of all ages across the life-course?

The urgency to address loneliness is paramount, impacting individuals, employers, communities, educators, and health professionals alike. Previous systematic reviews, notably constrained by their focus on older samples, have left a significant gap in understanding loneliness interventions across all age groups [6, 10–13]. Recognizing that meaningful social relationships are not only integral to individuals’ physical and mental health but also pivotal for workplace engagement and community cohesion, there is an immediate need to consolidate and analyse the current, diverse body of evidence. Tackling loneliness is an urgent priority to inform research, policy, and practice.

This rapid systematic review and meta-analyses aim to bridge these knowledge gap, offering insights into effective loneliness interventions to address the pressing question: What is the effectiveness of interventions aimed at alleviating loneliness in people of all ages?

## Data and methods

This rapid review was conducted following the Preferred Reporting Items for Systematic Reviews and Meta-Analyses guidelines [24] and guidance from the Cochrane Collaboration on rapid reviews [25]. The study protocol was registered with PROSPERO (CRD42023398520).

### Eligibility criteria

Studies from peer-reviewed journals and grey literature sources were eligible for inclusion if they met the following PICO (Population, Intervention, Control, and Outcome) criteria. Published on or after 2008, as requested by the review funder based on previously commissioned work. Eligible populations included all children and adults (no health or age restrictions), in a country within the Organisation for Economic Co-Operation and Development (OECD). Any intervention study, with or without a control group, was eligible if alleviation of loneliness was one of three or fewer intervention aims, and loneliness was assessed both pre- and post-intervention using a standardised quantitative measure. Furthermore, records must have been available in English and ﻿have sufficient detail to appraise study quality (conference abstracts and presentation slides were excluded). The restriction of three or fewer intervention aims was implemented to ensure that only studies that were designed and delivered with the aim to improve loneliness were included; conversely, this allowed studies that aimed to improve other aspects of health (weight loss, depressive symptoms) yet collected a large amount of outcome data to be excluded. The restriction to OECD countries aimed to minimise heterogeneity in populations and the context in which interventions were delivered. The implications of this geographical restriction were discussed with an expert panel who agreed that, given the common OECD member country focus on loneliness and social connection policy and indicators, the criteria would return an acceptable and more homogenous set of results.

### Search strategy

The search strategy had two arms: peer-reviewed literature and grey literature. Eligible peer-reviewed literature was identified by searching OVID MEDLINE, PsycINFO, and ERIC. Eligible grey literature was identified using electronic databases, specifically Google Scholar Advanced search, Social Science Research Network, Psych EXTRA and Social Care Online, and targeted searches of relevant websites. Grey literature searches were sorted by relevance and the first 500 records on each database reviewed. Searches were conducted in February 2023, restricted to 2008 onwards, and used free-text and MeSH (or APA mapping) terms as well as truncation and wildcards as appropriate in each database. The search strategy had two primary arms: ‘loneliness’ and ‘intervention’ (see Supplementary Text S1 and Table S1 for details and example).

### Study selection

The two arms of the search were managed separately. First, results from the three academic databases were combined using a reference software to remove all duplicates. Next, all title and abstracts were screened by the review team against the list of exclusion criteria using Rayyan [26]. Following rapid review guidance from the Cochrane Collaboration [25], two reviewers independently screened the first 25% of all records and discussed any conflicts to reach a final consensus decision. A single reviewer screened the remaining 75% of the records. In the second stage, the full text of all studies that passed the title-abstract screening stage was obtained. Once again, two researchers independently screened 25% of the full-text records and resolved any conflicts via discussion or consultation with a third member of the review team; a single reviewer screened the remaining 75%. To ensure that no evidence was missed or inadvertently included, a second reviewer confirmed all full-text decisions [25]. Reviewers recorded the reason for exclusion following a hierarchical list:

i. Non-English
ii. Inappropriate record type
iii. Non-OECD country
iv. No intervention
v. Loneliness not a primary intervention aim
vi. No validated/standardised measure of loneliness
vii. Did not measure loneliness pre- and post-intervention

These steps were replicated for the grey literature search. During protocol development, pilot search results identified a high number of studies—primarily clinical interventions—aiming to improve medical outcomes, whilst measuring loneliness as a secondary or tertiary outcome amongst 10 + other measures. Therefore, after discussion with an expert panel, we restricted eligibility criteria to interventions that targeted loneliness as a primary aim to minimise heterogeneity and ensure review findings had high fidelity of what works to improve loneliness.

### Data extraction

For all articles that met the inclusion criteria, a single reviewer independently extracted all study data. All extracted data were confirmed by a second member of the review team against the original record. The following data were extracted: record type (peer-reviewed paper or report), publication year, study sample (age; location; description including specific sex, ethnicity or health characteristics), study design (control group; randomisation; method of allocation), intervention (theme; description including frequency, length and context of intervention; duration of follow-up), loneliness data (standardised measure/scale deals; sample size, mean, and standard deviation pre- and post- intervention for intervention and control groups (and by any subgroup where reported)), and critical appraisal checklist. WebPlotDigitizer was used to obtain data presented in graphs and not available in tables [27]. Attempts were made to contact all authors for missing information on statistical estimates (means and standard deviations (SD)).

### Critical appraisal

﻿Two reviewers used the ‘What Works Centre for Wellbeing (WWCW) Quality Checklist: quantitative evidence of intervention effectiveness’ to independently appraise the quality of selected studies, developed by WWCW academics and the Office for National Statistics (ONS) following the Early Intervention Foundation (EIF) Standards of Evidence [28]. The checklist can be used across various study designs and assesses ten elements: fidelity, measurement, counterfactual, representativeness, sample size, attrition, equivalence, measures, analysis, and interpretation of findings (see Supplementary Table S2). Scores for each element are binary: 1 is affirmative and 0 constitutes ‘no’ or ‘not applicable’. Any conflicts were resolved by a third reviewer. Scores for each record were categorised as low (0–2), moderate (3–6), or high (7–10) levels of confidence [28]. Although the checklist is not intended as a full risk of bias assessment, however, it is a practical and validated tool that provides preliminary data on study quality for a range of non-randomised designs and is appropriate for a rapid review.

### Synthesis

A narrative synthesis following guidelines established by Popay et al.[29] was conducted, describing sample characteristics, intervention types, data extraction, and critical appraisal findings. Where possible, intervention characteristics including frequency, duration, format, and context/setting were included in the narrative synthesis to support quantitative. Interventions were coded by core theme and sub-theme, which were developed by analysing available data on intervention aims, activities, and settings. The most commonly reported data were pre- and post-intervention loneliness scores from those receiving the intervention. Therefore, we conducted random-effects meta-analyses of standardised mean differences (SMD), known also as Hedge’s *g* [30] for each theme (and sub-theme) to assess differences in loneliness from pre- to post-intervention using the meta and metaphor packages in R. Where data were not presented, we followed recommended Cochrane Collaboration approaches and calculated missing means and/or SD using minimum, maximum, median, interquartile ranges, and sample sizes where data were available (more detail on equations available [31, 32]). Any remaining missing SDs were imputed using summary statistic level imputation by loneliness measure (average SD for each measure) [31, 32].

Aggregate SMD effect sizes were reported for sub-themes with data from 4 + studies; 0.20, 0.50, and 0.60 correspond to small, medium, and large effect sizes, respectively [33]. Where possible due to sufficient number of studies reporting required data, supplementary meta-analyses tested for subgroup differences across main theme by age (<18y, 18–49y, 50+y) and region (Europe, North America, Other). The SMD is calculated using extracted means (m) and standard deviations (SD) as

1 $$SMD= \frac{{m}_{pre-intervention}-{m}_{post-intervention}}{{SD}_{pooled}}$$

In studies that reported mean and SD for both the intervention and control groups before and after the intervention, we conducted a second meta-analysis of difference in change scores following the same considerations as above. Where two intervention arms were presented against a single control group, we pooled the two intervention groups into one before including in the meta-analysis. Due to a smaller number of studies reporting estimates for intervention and control groups, meta-analyses were conducted for overall theme only. For each meta-analysis, heterogeneity was measured using the I2 statistic, in which > 75% indicates considerable heterogeneity [32]. To assess for publication bias, we used funnel plots and Egger’s regression intercept [34] to test for publication bias using (1) estimates from all pre–post-interventions groups (SMD) and (2) estimates from all mean change score differences in interventional and control group.

## Results

### Search results

**Fig. 1:**
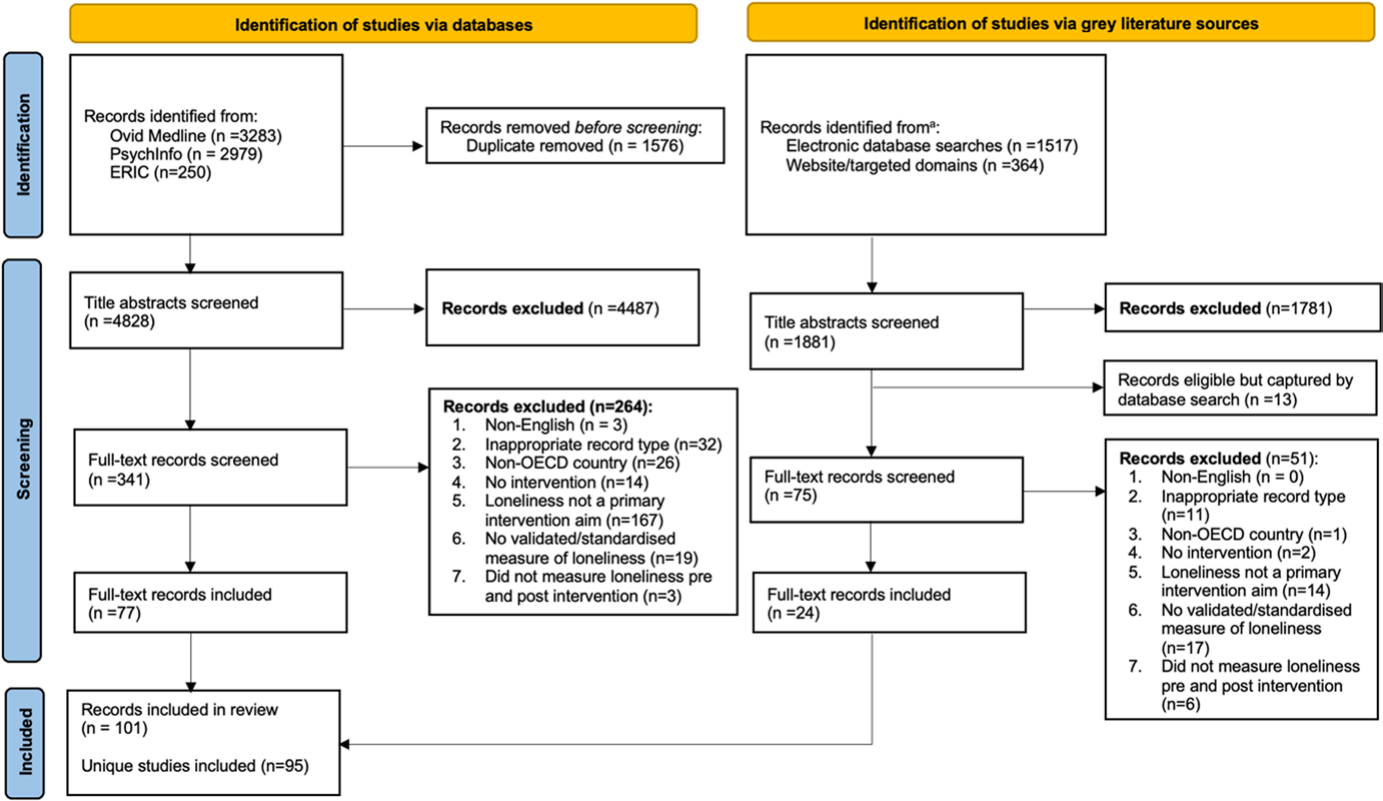
PRISMA flow diagram

The peer-reviewed literature search identified 6,512 electronic database records; 341 were selected for full-text screening and 77 met the inclusion criteria. The grey literature search identified 1,881 records from electronic database searches (*n* = 1,517) and websites or targeted domains (*n* = 364). Of the 75 full-text records screened, 24 met the inclusion criteria. The most common reason for exclusion was loneliness not being a primary intervention aim or the study not having a validated measure of loneliness. Duplicates between the peer-reviewed literature search and grey literature search were removed at the full-text screening stage. In total, data were extracted from 95 records; some records reported multiple intervention arms which were extracted separately, leading to 101 interventions included in the final sample for analysis (Fig. 1).

### Study characteristics

A summary of included studies is provided in Table 1. Peer-reviewed publications were most common (*n* = 79), with all other evidence coming from evaluation reports (*n* = 23). Approximately, half of studies examined older adults (aged ≥ 50; *n* = 56), a third examined adults aged 19 to 49 (*n* = 31), and a small proportion focussed on younger participants (aged < 18; *n* = 7), with three studies including participants from across the full life span (ages 8–83 [35]). Most studies were conducted in Europe and North America (*n* = 48 (51%) and *n* = 34 (36%), respectively), with a rise in publication rate in recent years (25% in 2021 and 16% in 2022). More than half of studies had no control group (*n* = 49; 52%) or randomisation (*n* = 54; 57%); the majority of those which did randomised participants individually (*n* = 23; 24%), whilst 14 used a wait-list control group (15%), and 4 used cluster randomisation (4%). The UCLA Loneliness Scale [36], ranging from 3 to 20 items, was the most common standardised loneliness measure in the sample (*n* = 66; 63%), followed by the De Jong Gierveld Scale [37] (*n* = 23; 22%) or a single-item direct measures of loneliness such as “How often do you feel lonely?” (*n* = 8; 8%).

**Table 1:** Study characteristics of each intervention (*n* = 101)

Study characteristics	*N* (%)
Evidence type (*n* = 95 studies)	
Peer-reviewed publication	75 (78.9)
Evaluation report	20 (21.1)
Age group (*n* = 97)^a^	
Under 18 s	7 (7.4)
Young and Mid-age Adults (19–49)	31 (32.6)
Older adults (50 +)	56 (58.9)
Full age range/life span	3 (3.2)
Country (*n* = 95 studies with unique data)	
Australia & New Zealand	7 (7.4)
North America	34 (35.8)
Europe	48 (50.5)
Central and South America	1 (1.1)
Asia	5 (5.3)
Control group (*n* = 95 studies)	
No	49 (51.6)
Yes	46 (48.4)
Randomisation (*n* = 95 studies)	
Individual randomisation	23 (24.2)
No randomisation nor wait-list	54 (56.8)
Wait-list control group	14 (14.7)
Cluster randomisation	4 (4.2)
Intervention theme and sub-theme (*n* = 101 interventions)^b^	
*Psychological interventions (n* = *23)*	23 (22.8)
Therapy	14 (60.9)
Other	9 (39.1)
*Social interaction (n* = *23)*	23 (22.8)
Arts/Music/Culture	8 (34.8)
Multiple activities	7 (30.4)
Other	8 (34.8)
*Social support (n* = *46)*	46 (45.5)
Befriending/Mentoring/Peer-support	20 (43.5)
Social prescribing/Connector Service	6 (13.0)
Knowledge/Skills development	11 (23.9)
Other	9 (19.6)
*Multiple themes (n* = *9)*	9 (8.9)
Multiple components	6 (66.7)
Strategic/grant-level study	3 (33.3)
Intervention format (*n* = 101 interventions)	
Individual	41 (40.6)
Group	40 (39.6)
Mixed	20 (19.8)
Loneliness measure (*n* = 104 studies)^c^	
Version of UCLA Scale	66 (63.5)
De Jong Gierveld Scale	23 (22.1)
Single-item measure	8 (7.7)
Other scale (4 scales)	7 (6.7)

^a^Two additional samples from mentee-mentor interventions

^b^% of sub-theme described as proportion within each theme

^c^Nine studies used multiple loneliness scales

### Key findings by theme

**Fig. 2:**
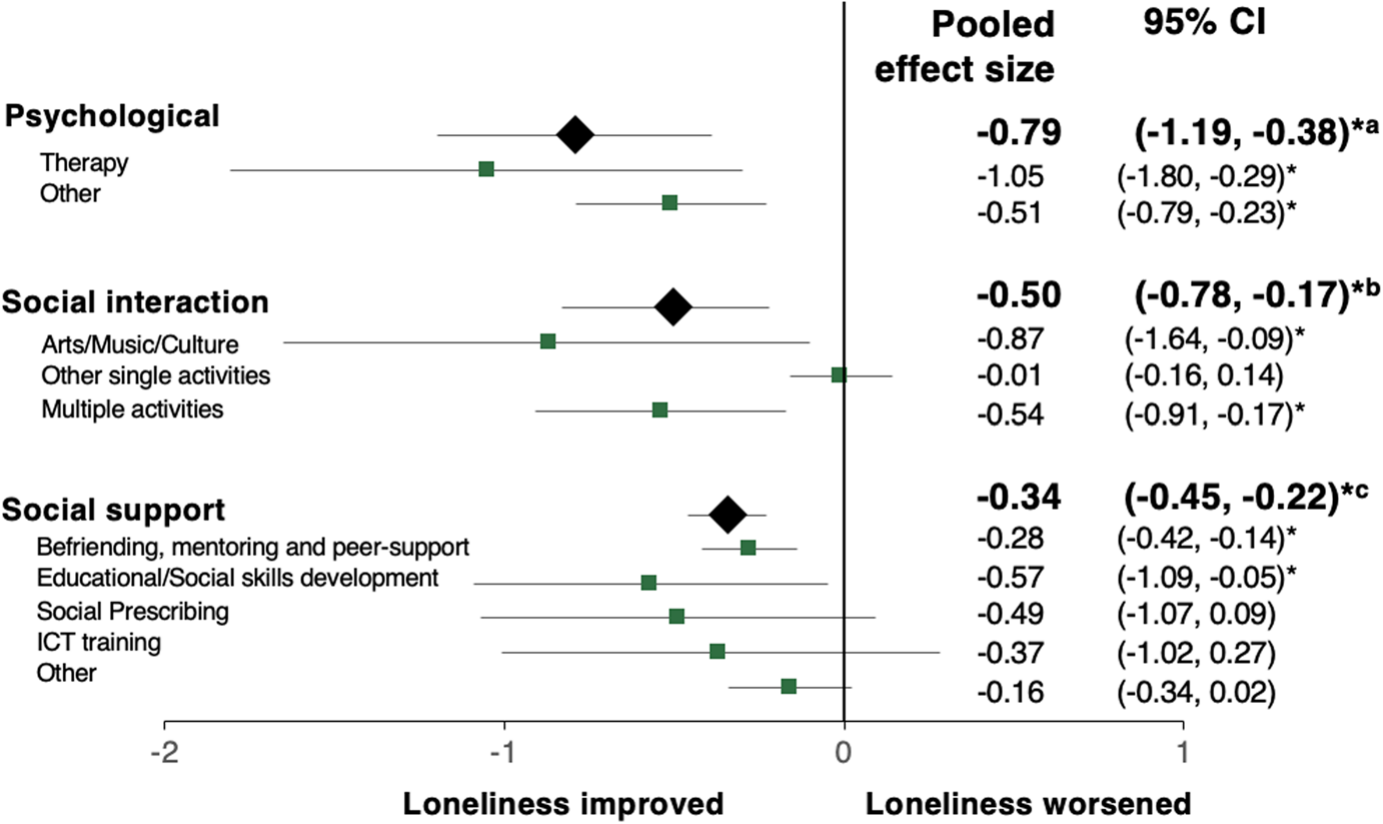
Summary of pooled effect sizes by theme and sub-theme

Interventions were categorised thematically during analysis into (1) psychological (*n* = 23), (2) social interaction (*n* = 23), and (3) social support (*n* = 46). Figure 2 describes a summary of the primary meta-analyses across sub-themes for psychological (− 0.79, 95%CI: [− 1.19, − 0.38]; large effect size), social interaction (− 0.50, 95%CI: [− 0.78, − 0.17]; medium effect size), and social support (− 0.34, 95%CI: [− 0.45, − 0.22]; small-medium effect size) groupings, ordered by pooled effect size; see subsequent sections for a breakdown by theme and sub-theme. Negative effect sizes demonstrate a reduction in loneliness score from pre- to post-intervention. Nine studies were cross-cutting and comprised two or more themes which could not be grouped in the other categories. An overview of study characteristics is provided in Table 1 and intervention core themes are described in Table 2. Supplementary Tables S4–S7 provide study-level detail on country of intervention, sample size and description, and intervention delivery.

**Table 2:** Breakdown of interventions by core theme

Theme	Description	Number
Psychological intervention	Interventions where the emphasis is on targeted non-pharmacological therapeutic support/treatment, often based on a psychological theory	23
Social interaction	Interventions where the aim is to reduce loneliness by increasing opportunities for social contact and growing an individual’s social relationships or network	23
Social support	Interventions that provide medium to longer-term and semi-/structured social support or equip individuals with the necessary skills to facilitate social connection	46
Multiple themes	Interventions with multiple components that span two or more core themes	9

### Theme 1: psychological

**Fig. 3:**
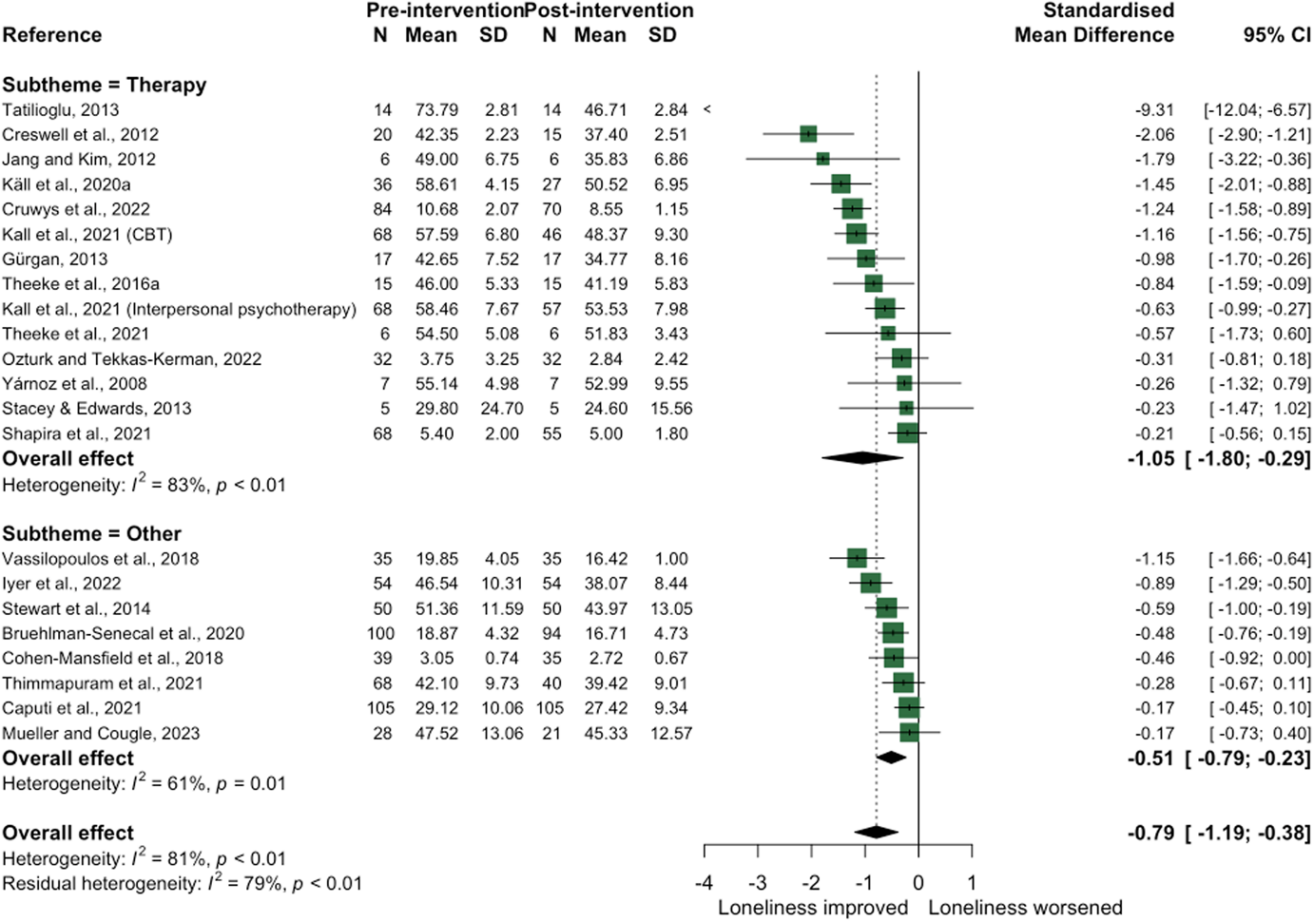
Forest plot demonstrating the effect of psychological interventions by sub-theme on change in loneliness from pre- to post-intervention

There were 23 psychological studies categorised in two sub-themes: (1) 14 structured therapy-based interventions and (2) nine ‘other’ approaches. All but one intervention provided sufficient information to be included in the meta-analysis, which indicated a large overall effect size of − 0.79 (95% confidence interval: [− 1.19, − 0.38]), albeit with a wide confidence interval. When aggregated by sub-theme, there was a very large SMD effect size for therapy-based intervention (− 1.05 [− 1.80, − 0.29]; heterogeneity I^2^ = 83%) and a moderate effect size for interventions classed as ‘other’ (− 0.51 [− 0.79, − 0.23]; I^2^ = 61%) (see Fig. 3).

**Fig. 4:**
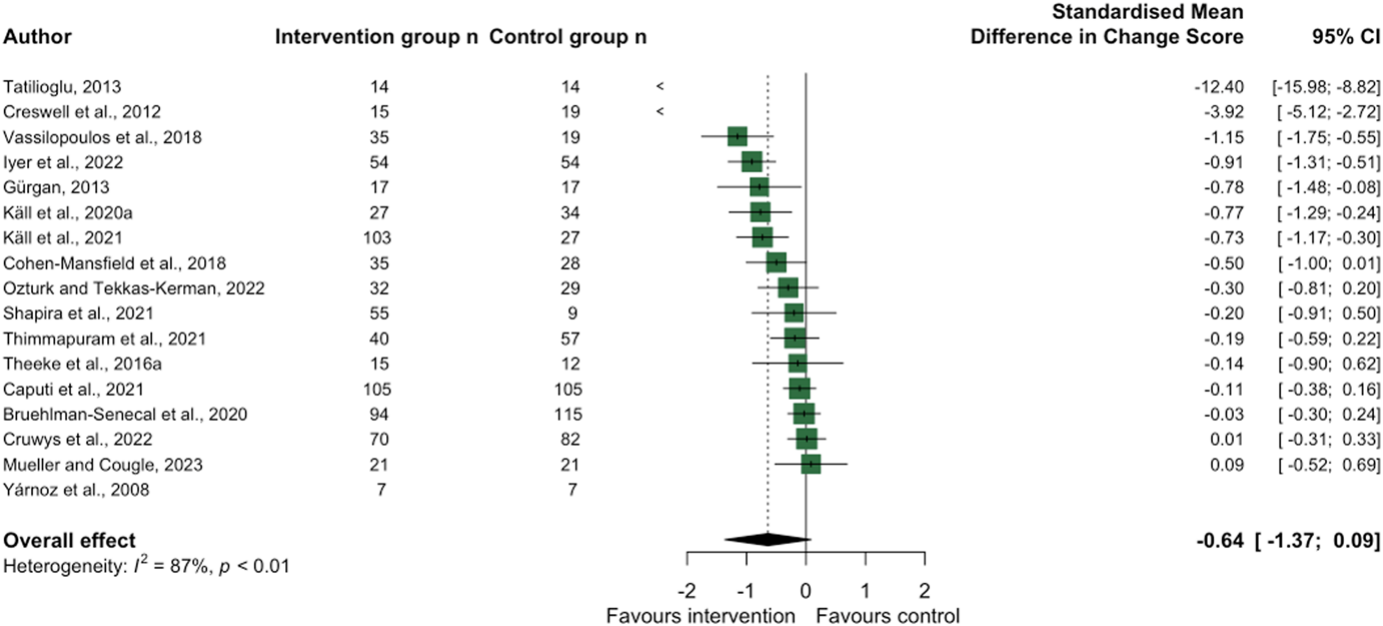
Forest plot demonstrating the effect of psychological interventions on change in loneliness from pre- to post-intervention between intervention and control group

Therapeutic approaches included cognitive behavioural therapy (CBT) [8, 9, 18, 38, 39], mindfulness for older adults [40], and group therapy approaches involving sand play [20], narrative therapy [41], laughter [17], and attachment-based therapy [42]. Studies included in the ‘other’ theme utilised psychological interventions to develop emotional and social skills amongst a range of target samples including older adults [43], adults with social anxiety disorder [44] and with adolescents (11–18 years) [45], and university students [46] (see Supplementary Table S4). Finally, sixteen studies also presented data from a control group (Fig. 4), which confirmed an overall large—albeit not statistically significant—effect of psychological interventions on loneliness (− 0.64 [− 1.37, 0.09]; I^2^ = 87%). When intervention characteristics were further investigated by dose or frequency, effectiveness was typically greater for interventions that lasted 1–2 months.

### Theme 2: social interaction

**Fig. 5:**
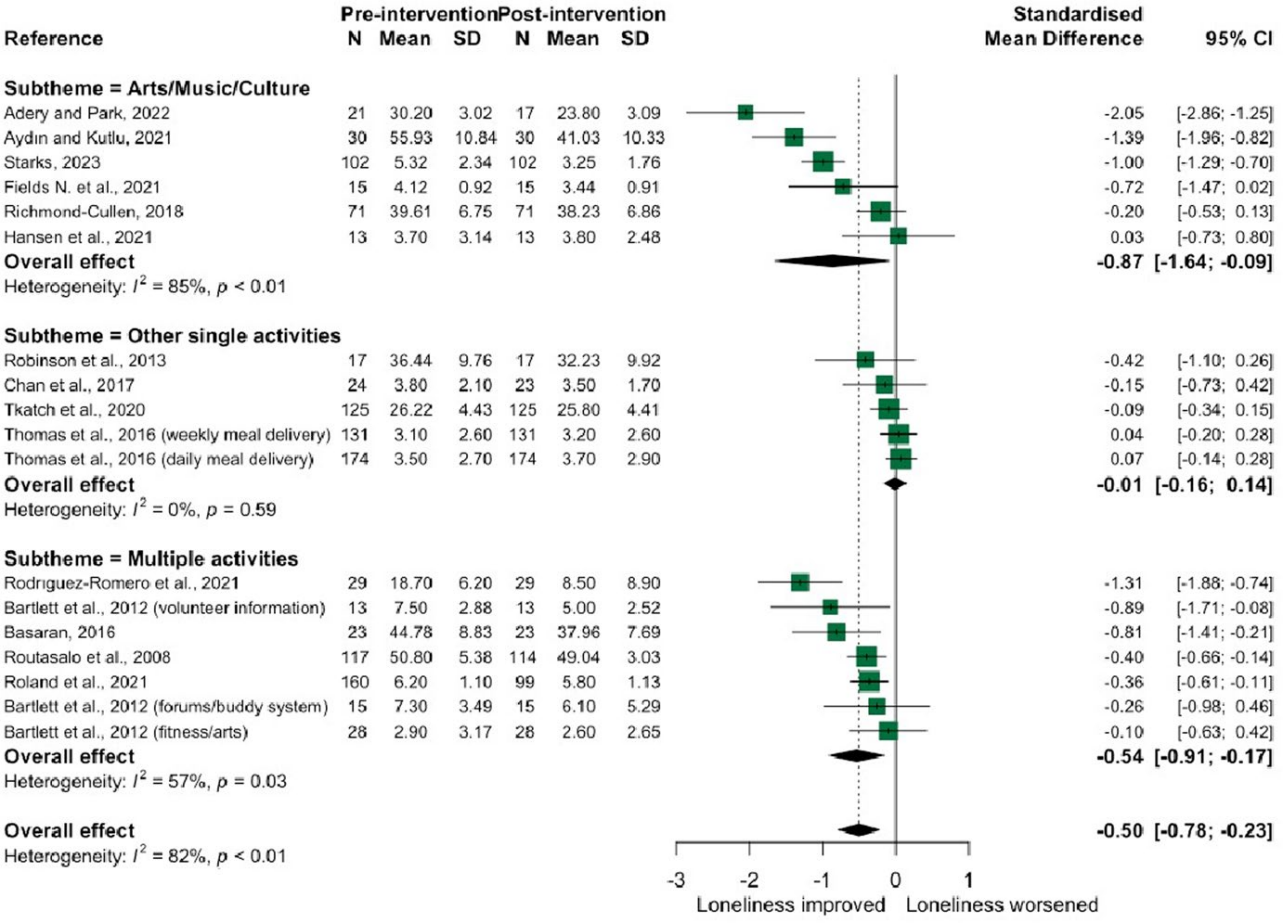
Forest plot demonstrating the effect of social interaction interventions by sub-theme on change in loneliness from pre- to post-intervention

From the 23 interventions (*n* = 19 studies) included in the social interaction theme, there were three sub-themes: art, music, and culture interventions (*n* = 8); ‘other single-themed’ (*n* = 8) comprising spiritual, physical activity, animal/robot interaction, food delivery, and online social groups interventions, and (3) interventions involving multiple activities (*n* = 7), such as community-level recreational activities (see Supplementary Table S5 for study details). Eighteen studies were included in the main meta-analysis examining difference in scores from pre- to post-intervention (Fig. 5), giving an overall moderate effect size of − 0.50 [− 0.78, − 0.23], I^2^ = 82%.

**Fig. 6:**
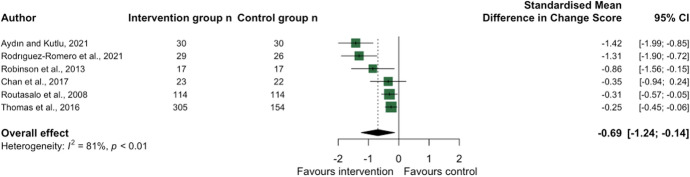
Forest plot demonstrating the effect of social interaction interventions on change in loneliness from pre- to post-intervention between intervention and control group

Art, music, or culture-based interventions produced a large overall effect size (− 0.87 [− 1.64, − 0.09]), with larger effect sizes emerging for interventions that lasted at least 1–2 months. There was no evidence to suggest that ‘single-themed’ social interaction interventions alleviated loneliness (− 0.01 [− 0.16, 0.14]); however, interventions involving multiple activities showed a moderate overall effect size (− 0.54 [− 0.91, − 0.17]). The latter included a programme of social health promotion in older adult participants, a programme connecting senior migrants to leisure activities and library services [47] and a 12-week programme of recreational activities aimed at female prisoners[48]. Only six studies from the social interaction theme evaluated loneliness in both intervention and control groups (Fig. 6), with a moderate-large overall effect size of − 0.69 [− 1.24, − 0.13]; statistical heterogeneity remained high (I^2^ 81%).

### Theme 3: social support

**Fig. 7:**
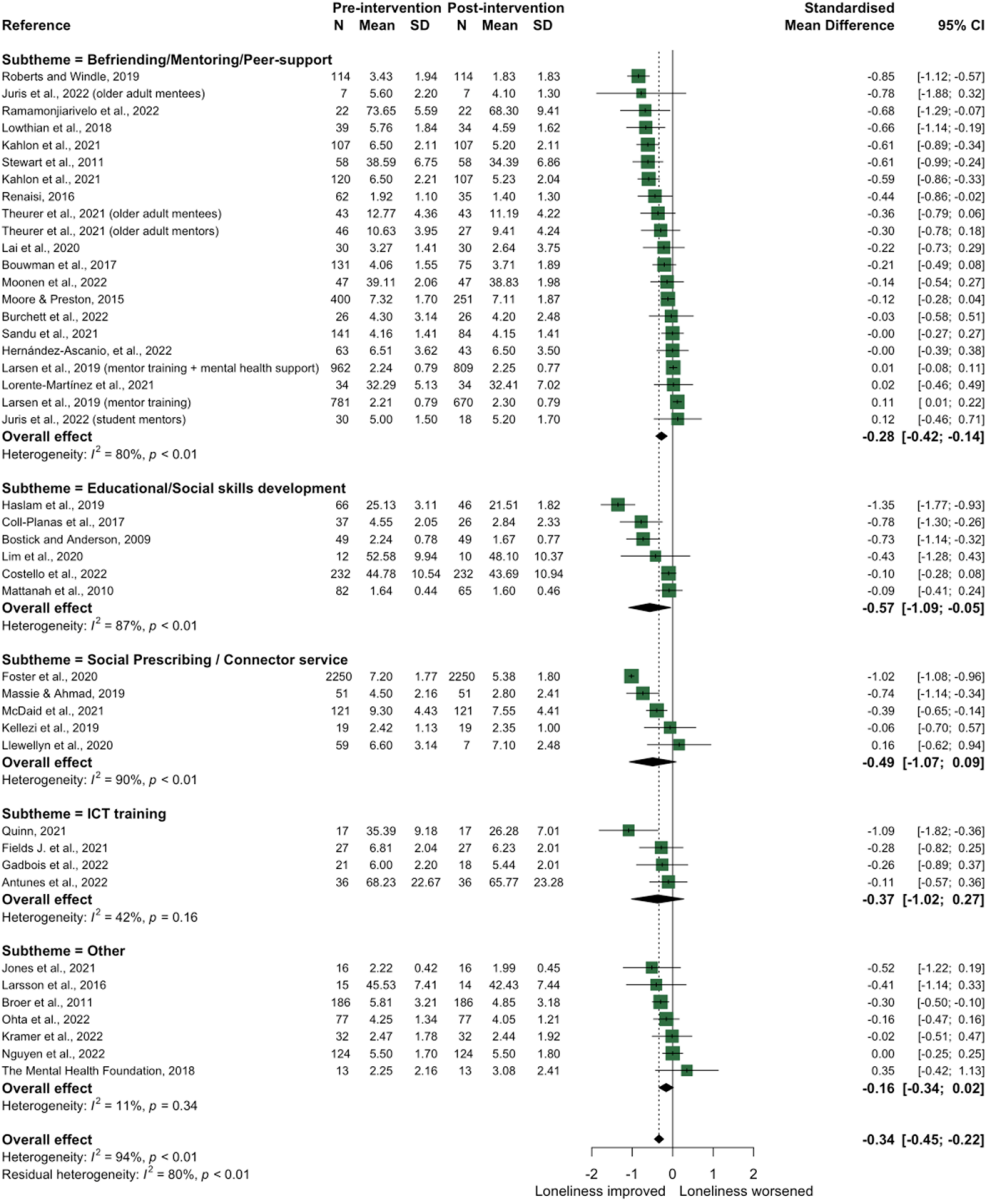
Forest plot demonstrating the effect of social support interventions by sub-theme on change in loneliness from pre- to post-intervention

**Fig. 8:**
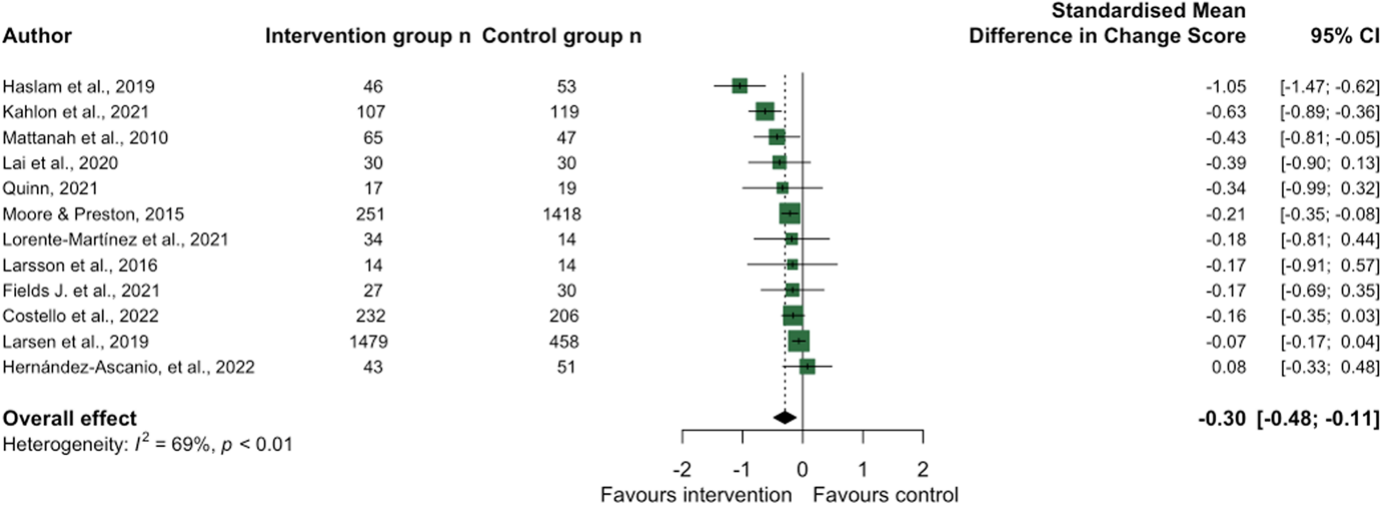
Forest plot demonstrating the effect of social support interventions on change in loneliness from pre- to post-intervention between intervention and control group

Social support was the most common theme, with 46 social support interventions across 45 studies aggregated into five sub-themes: (1) befriending, mentoring, and peer support (*n* = 20); (2) educational or social skills development (*n* = 6); (3) social prescribing (*n* = 6); (4) information and communications technology (ICT) training (*n* = 5); and (5) interventions classified as ‘other’ (*n* = 9) (see Supplementary Table S7 for study details). The latter comprised various activities delivered at the community level and in healthcare settings. Forty-one studies provided mean loneliness scores pre- and post-intervention and could be included in the main meta-analysis; the overall effect for social support interventions was − 0.34 [− 0.45, − 0.22] (Fig. 7). Due partially to the high number of studies and partially to many studies identifying low to moderate effect sizes, the precision of the 95% CI was higher than previous themes; however, statistical heterogeneity remained high (I^2^ 94%). Only 17 studies provided information on the change in loneliness for both the intervention group and control, with 12 studies included in the secondary meta-analysis; the overall effect size of the difference in change scores between groups was small (− 0.30 [− 0.48, − 0.11]; see Fig. 8).

There was a moderate overall effect size for befriending, mentoring, or peer-support-based interventions (− 0.28 [− 0.42, − 0.14]). Two studies presented loneliness data from both mentor and mentee groups who participated in the intervention, with mixed evidence. For example, loneliness decreased in both mentors and mentees in an intervention delivered with older adults living in a care home [49], whereas a mentoring scheme with university student mentors and older adult mentees reported no significant change in either group [50].

There were mixed results for educational or social skills development interventions, with an overall moderate effect size of − 0.57 [− 1.09, − 0.05]. The greatest improvement in loneliness was demonstrated by a 4-week social skills support intervention for adults with symptoms of depression [51]. All social prescribing interventions were UK-based, lasting between 2 and 9 months and primarily involved link worker models targeting adults with clinical needs [52–55] or experiencing/at risk of experiencing loneliness [56, 57]; however, there was no significant change in loneliness (− 0.49 [− 1.07, 0.09]). Finally, ICT training interventions (− 0.37 [− 1.02, 0.27]) targeting older adults (≥ 50 years) and interventions classified as ‘other’ (− 0.16 [− 0.34, 0.02]) found non-significant changes in loneliness post-intervention. The latter included interventions such as using an Amazon Echo personal voice assistant with older adults [58], a 4-month multi-tier intervention consisting of group and one-to-one goal-orientated social internet-based activities [59] and helping intellectually disabled adults increase their social networks [60] (see Supplementary Table S6).

### Multiple themes

**Fig. 9:**
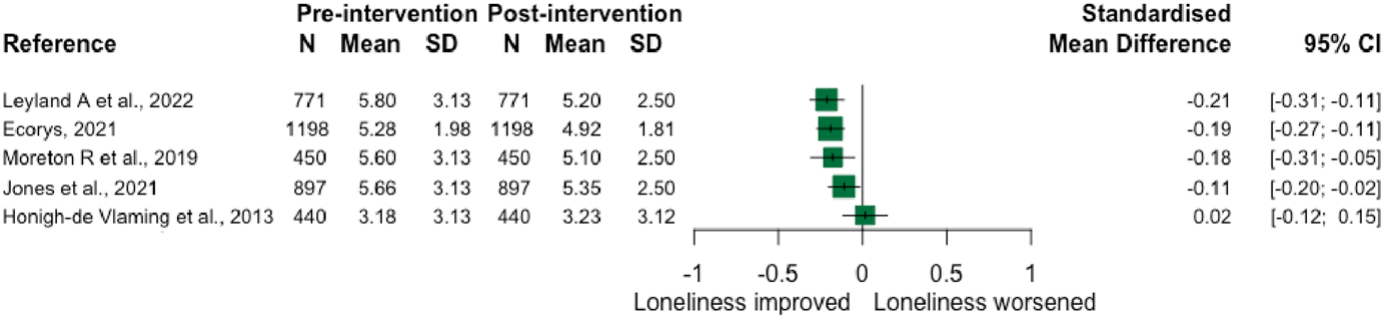
Forest plot demonstrating individual effects of multiple theme interventions on change in loneliness from pre- to post-intervention

Nine interventions involved activities that spanned two or more core themes. The majority were grey literature evaluation reports of national and city-wide programmes, largely aimed at older people (≥ 50), delivered following the UK’s national strategy [61] to tackle loneliness and social isolation in 2018. Four studies were included from The Ageing Better programme [62–66], which aimed to promote the active involvement of adults aged over 50 years in their communities. It involved multiple components including intensive one-to-one support, social prescribing/connector interventions, and social activities to promote social interaction (see Supplementary Table S7). Five studies reported mean loneliness scores pre- and post-intervention, with the four studies from The Ageing Better programme demonstrating improvements in loneliness over time (see Fig. 9) [62–64, 66]. Due to substantial differences in intervention (type, modality, delivery, context), no aggregate effect size was calculated; of note, all individual effect sizes were small (≤ 0.21; Fig. 9).

### Publication bias and sensitivity analyses

Publication bias is the failure to publish study results based on the direction or strength of the study findings [67]. There was no evidence of publication bias in the pre- and post-intervention meta-analysis of standardised mean differences, demonstrated by visual inspection of the funnel plot in Supplementary Figure S1 and the Egger’s test (*p* = 0.19). However, there was evidence of publication bias in the meta-analysis of differences in pre- and post-intervention loneliness scores between control and intervention groups (Supplementary Figure S1, Egger’s *p* < 0.001). Only 12 studies presented results stratified by subgroup characteristics; this included gender, age, socioeconomic status, ethnicity, caring status, and service use. Due to heterogeneity in reporting (different subgroups, prevalence of loneliness versus mean scores), it was not possible to synthesise and report this.

Using meta-analysis subgroup analyses, there was no difference in intervention effectiveness by region for psychological (*p* = 0.71), social support (*p* = 0.54) or social interaction (*p* = 0.35) interventions. Although there were no age group differences for social support (*p* = 0.75) or psychological (*p* = 0.70) interventions, subgroup analysis indicated that interventions in adults aged 19–49 (*n* = 3 studies; − 1.20 [− 2.72, 0.32]) may be slightly more effective than in adults aged 50 + (*n* = 12; − 0.38 [− 67, − 0.09]) (*p* = 0.02; Supplementary Figure S2).

### Quality assessment

A total of 63% of studies scored as high quality (*n* = 60; 7–10 points), 37% as moderate (*n* = 35; 3–6 points), and none as low quality (0%; 0–2 points). The lack of studies categorised as low quality suggests that the checklist (Supplementary Table S2) is likely to have overestimated overall quality, further supported by the binary scoring of each eligibility criteria. The proportion of studies satisfying the fidelity and measures criteria was high (*n* = 92; 97% each), demonstrating that most studies in this review provided sufficient detail on intervention designs and used a standardised, validated measure of loneliness published independently. Consistency between the reported results and discussion was high (*n* = 84; 88%) and 86% of studies (*n* = 82) presented results from statistical analysis. An ‘intent-to-treat’ design was used by 82% of studies, which included individuals who were lost to follow-up.

Failure to report on the representativeness of study samples was the most common reason for studies not meeting the representative criteria (*n* = 68; 72% scored positively). The requirement of a minimum sample size of 20 participants completing the measures pre- and post-intervention was only achieved by 69% of studies (*n* = 66). There is likely to be substantial attrition bias across study samples as almost half the sample did not describe differences between baseline respondent samples and those lost to follow-up or meet the low attrition criterion of 35% of participants completing pre- and post-measures (*n* = 48; 51%). As many studies did not have control groups, scores on equivalence (*n* = 37; 39%) and counterfactual (*n* = 36; 38%) criteria were low; therefore, it is difficult to attribute any changes in participant loneliness to the interventions themselves. A breakdown of interventions by quality assessment score is shown in Supplementary Figure S3.

## Discussion

Evidence from this review suggests that a wide range of interventions work to alleviate loneliness across age groups. There appear to be multiple approaches to combating loneliness, almost all of which target specific age groups or vulnerable populations. Overall, the quality of included studies was relatively high, with more grey literature studies achieving ‘moderate’ scores, compared to peer-reviewed articles (11% and 76% scored ‘high’, respectively). However, only half of the included studies used controlled study designs, which constitute the more robust evidence base consolidated in this review. Amongst these studies, the most effective interventions for reducing short-term loneliness were psychological approaches including structured support or therapeutic programmes that develop emotional and social skills as well as arts, music or culture activities which facilitate social interaction. There was some evidence indicating that intervention effectiveness was greater for interventions lasting at least 1–2 months and that young-middle aged adults may benefit more from social interaction interventions than older adults; however, there were insufficient study data to conduct a reliable investigation of intervention components in relation to effectiveness.

Our results are consistent with other reviews which report strong evidence that psychological interventions are effective at alleviating loneliness [14, 23, 68]. Notably, Hickin et al. [68] investigated only psychological randomised control trials (RCTs) and, similar to this review, found CBT was the most common type of therapy studied across the lifespan. The focus of the current review on studies in which the alleviation of loneliness was a key aim provides a more comprehensive depiction of the effectiveness of loneliness interventions across intervention types, settings, and population groups/throughout the life-course. Recent reviews of loneliness interventions have highlighted heterogeneity between studies as a major limitation [68, 69]. This review aims to reduce clinical heterogeneity in intervention type by synthesising meta-analysis at the sub-theme level; however, the substantial degree of both clinical and statistical heterogeneity across themes limits the generalisability of findings. Additionally, many other reviews include < 40 studies [14, 68–70], whereas the breadth of our inclusion criteria in terms of study sample, loneliness measure, and intervention type enabled us to increase the number of interventions included (*n* = 101) and thus the relevance of findings.

It was unsurprising to observe larger effect sizes when examining loneliness change from pre- to post-intervention group, compared to difference in loneliness changes in the control vs intervention groups. Greater effects in the former may be due to regression towards the mean [71], inclusion of poorer quality studies, selection bias in those who participate, or the adaptive function of loneliness, whereby loneliness may motivate reconnection with others and thus loneliness improves independent of any intervention received [23]. Therefore, interpretation of both meta-analyses results within each theme must be done with caution. For example, psychological interventions demonstrated the largest effect size when comparing pre- and post-intervention scores (− 0.79 [− 1.19, − 0.38]), but the effect size was partially attenuated when considering differences in control vs intervention group (0.64 [− 1.37, 0.09]). Conversely, the effect of social interaction interventions on loneliness was larger in the control vs intervention group meta-analysis (− 0.69 [− 1.24, − 0.14]) compared to the pre–post-meta-analysis (− 0.50 [− 0.78, − 0.17]). This evidence suggests that participation in structured group activities, in particular, arts, music or culture-based activities, alleviates loneliness indirectly, by creating opportunities for social contact and connection [72]. It was notable that the precision of the effect estimates for psychological intervention was larger in both pre–post-intervention as well as control vs intervention analyses compared to social interaction and social support interventions; this further highlights the need to better understand intervention effectiveness, and how this may differ across target sample (e.g. by age) and intervention components.

Heterogeneity, the degree of variability between studies, was identified in this review and is attributable to clinical, methodological, and statistical factors. Specifically, many studies were targeted to diverse participant groups across different countries and settings, and researchers employed various statistical methods, sometimes reporting analyses aside from the overall difference in loneliness pre- and post-intervention. Clinical heterogeneity across interventions was particularly notable, as there were substantial differences in intervention frequency, duration, and group size. Additional sources of heterogeneity between studies in this review are likely due to the large number of non-randomised studies and the subsequent presence of bias, methodological diversity, and the presence of confounding factors in the analysis. An in-depth exploration of the more nuanced sources of heterogeneity would require a full risk of bias assessment to be undertaken, followed by a sensitivity analysis that excludes high bias studies; however, this lies outside the scope of this review. Further investigation into the key components of interventions, their pathways to loneliness alleviation, and the circumstances in which they work best will help increase the generalisability of findings. Publication bias was low when comparing loneliness measures pre- and post-intervention; however, bias was identified for evidence that compared intervention and control arms, highlighted further by the smaller number of studies providing this robust study design (< 50 studies).

Rapid reviews can improve the clarity and accessibility of research evidence for policymakers and other knowledge users. This review has identified evidence on the preliminary effectiveness of interventions aimed at alleviating loneliness and provides a basis on which to plan targeted psychological and social interventions in the future. For interventions that reduce loneliness through structured psychological support, commissioners and policymakers should consider investments in larger-scale trials to replicate promising interventions at the larger population-level, providing there is sufficient evidence on intervention implementation, feasibility, and acceptability. Given heterogeneity in intervention delivery, particularly in social support and social interaction interventions, more pilot and early-stage studies are required to test for efficacy. This includes a wide range of one-to-one befriending, mentoring or peer-support schemes, as well as interventions involving ICT or social skills development and single-group activities that aim to alleviate loneliness by growing an individual’s social network.

Commissioners and policymakers should ensure that policy and programme design build in time and funding for robust evaluation designs that use control groups. Wait-list control designs and the use of propensity score matching may offer a practical and ethically viable alternative to RCTs to evaluate the effectiveness of social support and psychological interventions. Design of primary research should address known knowledge gaps for specific intervention types and populations. Key gaps identified in this review include evidence on interventions that improve connectivity, including digital skills programmes, workplace interventions, and what works to alleviate loneliness for particular at-risk groups, such as ethnic minorities and LGBTQ + individuals. Another important consideration concerns the replicability of studies as most controlled studies were not carried out in the UK and may not be replicated in a UK context.

Given the importance of context in the design and effectiveness of loneliness interventions, theory-based and process evaluations provide evidence on how interventions work and in what circumstances. Whilst not possible in this review due to insufficient reporting of intervention components and the scope of the review, methodological approaches such as Intervention Component Analysis (ICA) and Qualitative Comparative Analysis (QCA) can help identify critical components, mechanisms and causal pathways of successful and unsuccessful loneliness interventions, where there is substantial clinical and statistical heterogeneity [73]. Their use should be pre-specified in the review protocol. Realist review approach and better reporting of subgroup differences in effectiveness would allow a better understanding of what works and for who. Research funders should support research to explore the context and implementation of interventions, to assess potential mechanisms and pathways of action of successful loneliness interventions.

### Strengths and limitations

This review adhered to rigorous systematic guidance from the Cochrane collaboration on rapid reviews [25], including registering a protocol and conducting a comprehensive search of multiple academic databases, and 19 grey literature sources. Careful consideration was used to minimise missing data (contacting authors, SD imputations, medians, etc.) for meta-analyses. Review inclusion criteria were broad in terms of study sample, loneliness measure, and intervention, enabling a wide range of evidence to be included, increasing the generalisability of the findings. The focus on studies where the alleviation of loneliness was one of three or fewer aims is a major strength as it limits search results to interventions that intentionally target loneliness and therefore can provide a more accurate depiction of what works to improve loneliness.

Nonetheless, there are several limitations. Firstly, only English-language studies were included, which may introduce bias due to missing evidence from non-English-speaking OECD-based countries. Due to the rapid nature of the review, only three academic databases were screened, and secondary screening of reference lists from included studies was not conducted. In addition, studies where loneliness was one of more than three primary outcomes were excluded, increasing the risk that potential evidence on loneliness effectiveness was missed. However, pilot searching indicated these were primarily medical interventions that collected loneliness alongside many additional outcomes; often the effect of the intervention on loneliness was therefore small given the intervention had not been designed to target this outcome. Although the impact of excluding these interventions may have resulted in larger effect sizes, this decision reduced some heterogeneity and enabled synthesis of the most relevant evidence from a policy perspective. The quality of some reports identified from grey literature searches was poor; overall, the high number of studies without a control group limits the overall quality of studies included in the review.

Another limitation concerns the theoretical basis for intervention development which is underdiscussed in the included studies. This limits the ability to advance theoretical discussion and conceptualisation regarding definitions of loneliness, and the processes and mechanisms that drive improvements in loneliness for different populations and contexts. In addition, the loneliness measures used across studies capture the frequency of loneliness and do not allow for an analysis of intervention effectiveness in relation to the severity of loneliness over time.

Whilst the quality checklist used was appropriate given the rapid nature of the review and the variation in study design, its use of binary responses (yes; no/can’t tell) does not provide sufficient context on some key appraisal criteria and overestimates certain sub-items of the tool (fidelity, measures, etc.). In a full systematic review, the use of the RoB-2 and ROBINS-I tools would provide a more granular and systematic picture of study bias and a clearer overall assessment of the body of evidence for loneliness alleviation. To manage review scope and reduce intervention heterogeneity, only studies with a primary aim of reducing loneliness were included in the review. However, the majority of excluded studies that measured loneliness as a secondary or tertiary aim tended to be clinical interventions. We hypothesise that if these studies had been included, overall effect sizes would be lower given that interventions that do not directly target loneliness (targeting sleep or other clinical symptoms) are unlikely to be effective at decreasing loneliness.

Finally, approximately half of the studies included in the review did not have a control group to compare intervention effectiveness. Control groups are particularly important as factors independent to the intended intervention (e.g. changes in daily routine, bias due to participant expectations, external life circumstances) may have impacted the post-intervention measurement of loneliness [74]. Consideration of both single pre–post-intervention scores and comparing pre–post-differences between control and intervention groups in this review mitigated study design weaknesses; results were comparable, albeit showing slightly weaker effectiveness in studies that compared the two groups. Future intervention research must make every effort to include a control group for comparison, whilst future reviews on such topics should consider restricting criteria to study designs with a control group only. Furthermore, the Egger test indicated there was publication bias in studies that included a control group, further highlighting the need for publication of studies with a control group regardless of intervention effectiveness.

## Conclusions

The highest quality evidence for effectively reducing loneliness was found for psychological and social-interaction-based interventions. Therapy-based interventions and interventions involving arts, music, and cultural activities were especially effective, as the evidence suggests social skills building through targeted psychotherapy or tailored social exchanges can significantly reduce loneliness. The most effective interventions for reducing short-term loneliness were structured psychological approaches that provide therapeutic support and develop emotional and social skills and activities which facilitate social inclusion. The use of randomisation and control groups in future research can provide stronger findings on the impacts of support-themed interventions and address exiting knowledge gaps for specific intervention types and target populations more broadly.

## Supplementary Information

Below is the link to the electronic supplementary material.

Supplementary file1 (DOCX 353 KB)

## Data Availability

Not applicable.
