# Supplementary material for: What works to reduce loneliness: a rapid systematic review of 101 interventions
Source: J Public Health Policy. 2025 Mar 6;46(2):245–68. doi: 10.1057/s41271-025-00561-1 (PMC12119363; doi:10.1057/s41271-025-00561-1)
Supplement: Supplementary file 1 — Supplementary file1 (DOCX 353 KB) [file 41271_2025_561_MOESM1_ESM.docx]

**Supplementary Materials**

**Supplementary Text S1.** Summary of search strategy

**Supplementary Table S1.** Example of Ovid MEDLINE search

**Supplementary Table S2.** What Works Centre for Wellbeing (WWCW) Quality Checklist: Quantitative evidence of intervention effectiveness (checklist for study critical appraisal)

**Supplementary Table S3.** Breakdown of included studies by critical appraisal score

**Supplementary Table S4.** Characteristics of each intervention in the Psychological interventions theme (n=23)

**Supplementary Table S5.** Characteristics of each intervention in the Social Interaction theme (n=23)

**Supplementary Table S6.** Characteristics of each intervention in the Social Support theme (n=46)

**Supplementary Table S7.** Characteristics of each intervention in ‘Multiple themes’ (n=9)

**Supplementary Figure S1.** Funnel plot to assess publication bias in included studies from a) pre- and post-intervention changes in intervention group and b) differences in pre- and post-intervention loneliness scores between control and intervention groups.

**Supplementary Figure S2.** Subgroup meta-analysis of age differences in social interaction interventions (n=15 studies)

**Supplementary Text S2.** Reference list of studies included in review

**Supplementary Text S1.** Summary of search strategy

Study inclusion criteria

Studies were eligible for inclusion if they met the following criteria:

1. Any intervention delivered directly to individuals where the primary aim is to alleviate loneliness (if there are multiple primary aims, it must be one of three or fewer);
2. Intervention must be based in an OECD country;
3. Loneliness must be measured using a validated and standardised measure;
4. Loneliness must be measured before and after intervention (pre/post design);
5. English language

The exclusion criteria included:

1. Intervention where alleviating loneliness is a secondary aim or one of 4+ primary aims;
2. Intervention is based in a non-OECD country;
3. Do not use validated and a standardised measure of loneliness (including qualitative ascertainment);
4. Loneliness not measure before and after the intervention;
5. Non-English language;
6. Medical-based intervention (i.e. pharmaceutical) or delivered within a hospital setting.
7. Inappropriate record type (i.e. news article, book, dissertation)

Search strategy

The search strategy consisted of two arms: traditional academic databases and grey literature. Both arms combined two key constructs ‘loneliness’ and ‘intervention’ using Boolean operators, truncation symbols and MeSH terms/mapped subject headings (see Figure 1). Truncation symbols enabled various spellings of a given phrase to be included; for example, ‘program*’ would capture ‘programme”, ‘program’, ‘programe’, ‘programs’, etc.

Searches were conducted across three academic databases (Ovid Medline, ERIC, PsychInfo) using keywords and MeSH terms/mapped subject headings and were restricted to 2008 onwards. Grey literature searches were conducted across the electronic databases and websites/online repositories listed in Table S1 below.

**Supplementary Table S1. Example of Ovid MEDLINE search**

| 1 | (Loneliness or Loneliness assessment or "At risk for loneliness").mp. |
| --- | --- |
| 2 | Loneliness/ or Loneliness.mp. |
| 3 | loneliness.mp. or exp Loneliness/ |
| 4 | intervention*.mp. or Psychosocial Intervention/ [mp=title, book title, abstract, original title, name of substance word, subject heading word, floating sub-heading word, keyword heading word, organism supplementary concept word, protocol supplementary concept word, rare disease supplementary concept word, unique identifier, synonyms] |
| 5 | (program or programs or programme or programmes or programes).mp. or Program Evaluation/ [mp=title, book title, abstract, original title, name of substance word, subject heading word, floating sub-heading word, keyword heading word, organism supplementary concept word, protocol supplementary concept word, rare disease supplementary concept word, unique identifier, synonyms] |
| 6 | evaluation.mp. or Evaluation Study/ [mp=title, book title, abstract, original title, name of substance word, subject heading word, floating sub-heading word, keyword heading word, organism supplementary concept word, protocol supplementary concept word, rare disease supplementary concept word, unique identifier, synonyms] |
| 7 | 4 or 5 or 6 |
| 8 | 3 and 7 |
| 9 | limit 8 to yr="2008 -Current" |

**Supplementary Table S2. What Works Centre for Wellbeing (WWCW) Quality Checklist: Quantitative evidence of intervention effectiveness (checklist for study critical appraisal)**

The checklist below is from the quality checklist for quantitative evidence of intervention effectiveness. In a previous review, WWCW developed a scoring system to provide an indication of overall level of confidence in the design, conduct and reporting of the study. The 10 elements of the checklist can be scored either 1 (yes) or 0 (no, can’t tell or N/A). The total score can be used to assign each study an overall level of confidence of low (0-2), moderate (3-6) or high (7-10).

| **Question** | **Element** | **Response options** |
| --- | --- | --- |
| Was the evidence well-designed? | Fidelity:  • The extent to which the intervention was delivered with fidelity is clear – i.e., if there is a specific intervention which is being evaluated, this has been well reproduced. | Yes (1)  No (0)  Can’t tell (0)  N/A (0) |
|  | Measurement:   - The measures are appropriate for the intervention’s anticipated outcomes and population. - Participants completed the same set of measures once shortly before participating in the intervention and once again immediately afterwards. - An ‘intent-to-treat’ design was used, meaning that all participants recruited to the intervention participated in the pre/post measurement, regardless of whether or how much of the intervention they received, even if they dropped out of the intervention (this does not include dropping out of the study - which may then be regarded as missing data). | Yes (1)  No (0)  Can’t tell (0)  N/A (0) |
|  | Counterfactual:   - Assignment to the treatment and comparison group was at the appropriate level (e.g., individual, family, school, community). - The comparison condition provides an appropriate counterfactual to the treatment group. Consider: - Participants were randomly assigned to the treatment and control group through the use of methods appropriate for the circumstances and target population OR sufficiently rigorous quasi-experimental methods (regression discontinuity, propensity score matching) were used to generate an appropriately comparable sample through non-random methods. - The treatment and comparison conditions are thoroughly described. | Yes (1)  No (0)  Can’t tell (0)  N/A (0) |
| Was the study carried out appropriately? Including appropriate sample | Representative:  • The sample is representative of the intervention’s target population in terms of age, demographics and level of need. The sample characteristics are clearly stated.  • There is baseline equivalence between the treatment and comparison group participants on key demographic variables of interest to the study and baseline measures of outcomes (when feasible). | Yes (1)  No (0)  Can’t tell (0)  N/A (0) |
|  | Sample size:  • The sample size is sufficiently large to test for the desired impact. This depends most importantly on the effect size, however a suggestion could be, for example, that a minimum of 20 participants have completed the measures at both time points within each study group. | Yes (1)  No (0)  Can’t tell (0)  N/A (0) |
|  | Attrition:   - A minimum of 35% of the participants completed pre/post measures. Overall study attrition is not higher than 65%. - The study had clear processes for determining and reporting drop-out and dose. Differences between study drop-outs and completers were reported if attrition was greater than 10%.   The study assessed and reported on overall and differential attrition. | Yes (1)  No (0)  Can’t tell (0)  N/A (0) |
|  | Equivalence:   - Risks for contamination of the comparison group and other confounding factors have been taken into account and controlled for in the analysis if possible. - Participants were blind as to their assignment to the treatment and comparison group.   There was consistent and equivalent measurement of the treatment and control groups at all points when measurement took place. | Yes (1)  No (0)  Can’t tell (0)  N/A (0) |
|  | Measures:   - The measures used were valid and reliable. This means that the measure was standardised and validated independently of the study, and that the methods for standardisation were published.   Administrative data and observational measures may also have been used to measure programme impact, but sufficient   - Information was given to determine their validity for doing this. - Measurement was independent of any measures used as part of the treatment. - In addition to any self-reported data (collected through the use of validated instruments), the study also included assessment information independent of the study participants (e.g., an independent observer, administrative data etc) | Yes (1)  No (0)  Can’t tell (0)  N/A (0) |
| Was the analysis appropriate? | • The methods used to analyse results are appropriate given the data being analysed (categorical, ordinal/ratio, parametric/non-parametric, etc.) and the purpose of the analysis.  • Appropriate methods have been used and reported for the treatment of missing data. | Yes (1)  No (0)  Can’t tell (0)  N/A (0) |
| Is the evidence consistent? | - Are the findings made explicit? - Is there adequate discussion of the evidence both for and against the researcher’s arguments? - Has the researcher discussed the credibility of their findings (e.g., triangulation, respondent validation, more than one analyst)?   • Are the findings discussed in relation to the original research question? | Yes (1)  No (0)  Can’t tell (0)  N/A (0) |

**Supplementary Table S3. Breakdown of included studies by critical appraisal score**

|  | **N (%)** |
| --- | --- |
| **Overall summary score** |  |
| Low (0-2) | 0 (0%) |
| Moderate (3-6) | 35 (37%) |
| High (7-10) | 60 (63%) |
| **Element** |  |
| Fidelity | 92 (97%) |
| Measures | 92 (97%) |
| Consistency | 84 (88%) |
| Analysis | 82 (86%) |
| Measurement | 78 (82%) |
| Representative | 68 (72%) |
| Sample size | 66 (69%) |
| Attrition | 48 (51%) |
| Equivalence | 37 (39%) |
| Counterfactual | 36 (38%) |

**Supplementary Table S4. Characteristics of each intervention in the Psychological interventions theme (n=23)**

| **Authors** | **Country** | **Sample size ^a^ and description** | **Intervention description** |
| --- | --- | --- | --- |
| **THERAPY (N=14)** | | | |
| Creswell et al., 2012 | USA | n= 34 older community-dwelling adults | Mindfulness Based Stress Reduction Program; 8x 120-min group sessions weekly, a day-long retreat in the sixth or seventh week, and 30-min of daily home mindfulness practice |
| Cruwys et al., 2022 | Australia | n= 152 young people seeking mental healthcare | 5x 75 min group-based psychotherapy sessions aiming to build group-based belonging, max group size 5 |
| Gürgan, 2013 | Turkey | n= 34 university students | 11x 2hr weekly group psychological counselling sessions focusing on developing communication skills, social awareness, and emotional awareness |
| Jang and Kim, 2012 | South Korea | n= 11 migrant adult women in international marriages | 10x 1.5hr weekly Kalffian sandplay therapy (Kalff, 2003/1980); participants created a scene in a sand tray in silence and told the group of the emotions, physical sensations, memories and ideas they felt while making the sand scene |
| Käll et al., 2020a | Sweden | n= 61 adults who are suffering/experiencing distress as a result of loneliness (as assessed by the participant) | 8x weekly internet-based CBT online modules; each module revolved around themes of loneliness and involved a homework assignment for which participants received feedback from their paired therapist |
| Käll et al., 2021 (two intervention arms) | Sweden | n= 130 adults who are suffering/experiencing distress as a result of loneliness (as assessed by the participant) across two intervention arms | First intervention arm: internet-based CBT. This consisted of 9 modules that contained text, pictures, and interactive assignments related to the theme of loneliness and how to deal with it. Participants received weekly feedback on their work by their assigned therapist for 10-weeks intervention duration.  Second intervention arm: internet-based interpersonal psychotherapy. This consisted of 9 modules that contained text, pictures, and interactive assignments related to the theme of loneliness and how to deal with it. Participants received weekly feedback on their work by their assigned therapist for 10-weeks intervention duration. |
| Ozturk and Tekkas-Kerman, 2022 | Turkey | n= 61 nursing students | 8x online group 45min laughter therapy sessions (2/week for 4 weeks) consisting of clapping/warm-up exercises, deep breathing exercises, childlike playfulness and laughter exercises |
| Shapira et al., 2021 | Israel | n= 64 older community dwelling adults living on their own | 7x online sessions over 3.5 weeks consisting of guided online group discussions, mindfulness and CBT techniques |
| Stacey & Edwards, 2013 | England | n= 5 adult men with a mild learning disability | 8x 2hr narrative therapy group sessions examining loneliness, causes, solutions and developing personalised plans |
| Tatilioglu, 2013 | Turkey | n= 28 university students | 12x weekly online cognitive behavioural therapy via MSN messenger with weekly homework (material included explanations on loneliness, how to communicate better, and changing cognitive structures). |
| Theeke et al., 2016a | USA | n= 27 chronically ill older adults | 5x weekly 2hr group CBT-based sessions aiming to facilitate change in cognitive perspective; topics included belonging, relationships, socialization in community, challenges of loneliness, and meaning of loneliness |
| Theeke et al., 2021 | USA | n= 6 adult survivors of stroke | 5x weekly 2hr group CBT-based sessions aiming to facilitate change in cognitive perspective; topics included belonging, relationships, socialization in community, challenges of loneliness, and meaning of loneliness |
| Yárnoz et al., 2008 | Spain | n= 14 long-term divorced parents | 8-months of weekly 2hr group sessions (attendance on voluntary basis) delivering an attachment-based intervention for separated or divorced parents; aim was to share narratives discuss the events, the self, and the relationship that contributed to adapting better to being divorced. |
| **OTHER (N=9)** |  | | |
| Bruehlman-Senecal et al., 2020 | USA | n= 209 recent first-year college students | 4-weeks access to a smartphone app with cognitive and behavioural exercises when starting college, control group allowed access following a 4-week wait period. |
| Caputi et al., 2021 | Italy | n= 210 fourth and fifth graders | 5 weekly class-based Theory of Mind training sessions, each lasting 50 minutes with two mentalistic stories (i.e. discrepancy in belief between characters focusing on persuasion, misunderstanding, white lie, irony/sarcasm, contrary emotions) and a group discussion and language exercises about the stories |
| Cohen-Mansfield et al., 2018 | Israel | n= 63 older community-dwelling adults | iSOCIAL intervention which consisted of 1) identifying individual barriers, 2) up to ten individual meetings with an activities counsellor and 3) up to seven group sessions with participants to provide opportunities to practice social skills |
| Iyer et al., 2022 | USA | n= 108 high-school students | 28-day Self-Care programme; participants used guided tools to relax/meditate/affirm/breathe/rejuvenate/self-observe for 15 min/day and joined 4x weekly 30-min webinars that focused on managing stress, building a positive mind map, aligning with the daily circadian rhythm to improve sleep quality, and setting goals with self-observation |
| Mueller and Cougle, 2023 | USA | n= 42 adults with social anxiety disorder | 4x weekly online sessions psychoeducation expressive writing, social skills learning, exposure introduction and planning, exposure follow-up) with 2x additional online practice sessions each week; aimed at 'Building Closer Friendships' by reducing fear of intimacy |
| Stewart et al., 2014 | USA | n= 50 university students | 6x biweekly 2hr drop-in sessions with a therapy dog in student halls; informal group-based sessions involved petting the dog, hugging the dog, giving the dog treats, and brushing the dog's coat. |
| Thimmapuram et al., 2021 | USA | n= 97 physicians and advance practice providers | 4-weeks of daily heartful meditation practices in the morning and before going to bed (each practice consisted of 6-minute audio guiding participants to gradually relax their body and rest their attention in their heart space) |
| Travers and Bartlett, 2011 | Australia | n= 113 older community-dwelling adults | 3-months of daily listening to a 1hr radio programme specifically focused on the era from which older people grew up (1920s-1950s) broadcasting music, serials, and segments designed to address depression and loneliness in older adults |
| Vassilopoulos et al., 2018 | Greece | n= 54 6th grade school students | 5x weekly 1.5hr group sessions aimed to reduce fears/concerns about secondary school; session themes included social skills, cognitive restructuring, and conflict resolution |

^a^ Sample size represents number of participants who responded to post intervention loneliness measures across intervention and control groups

**Supplementary Table S5. Characteristics of each intervention in the Social Interaction theme (n=23)**

| **Authors** | **Country** | **Sample size ^a^ and description** | **Intervention description** |
| --- | --- | --- | --- |
| **ARTS/MUSIC/CULTURE (N= 8)** | | | |
| Adery and Park, 2022 | USA | n= 17 adults with ﻿schizophrenia-spectrum conditions | ﻿8x weekly 1hr group choral rehearsals |
| Apteligen, 2021 | UK | n= 230 female carers experiencing or at risk of low wellbeing and loneliness and/or social isolation | 9-week long group music-making activity aimed at female carers. Music creation projects included singing, song writing, learning an instrument, and digital music production. |
| ﻿Aydın and ﻿Kutlu, 2021 | Turkey | n= 60 older community-dwelling adults living on their own | 1.5hr clay sessions weekly for 6 weeks, participants arranged into 3 groups of 10 |
| Brown et al., 2019 | England | n= 18 older people experiencing or at risk of, experiencing social isolation and/or loneliness | A group of older people meet monthly (five-set sessions) for around 2 hours. Activities include sharing cake, socialising, and a heritage presentation and sometimes other heritage activity (e.g., handling objects, and/or visiting an associated exhibition or installation) |
| Fields N et al., 2021 | USA | n= 15 older adults in a residential care setting | 3x 10-minute sessions with a social robot engaging older adults in participatory arts (e.g. Shakespearean text, etc.) |
| Hansen et al., 2021 | Canada | n= 13 older community-dwelling adults | The Moving Connections Project; 4x 3.5hr choreography-based dance session held over 4 consecutive days |
| Richmond-Cullen, 2018 | USA | n= 71 older community-dwelling adults | 10x weekly or biweekly 2hr art sessions at 14 senior community centres as part of an artist in residence program; art type varied by artist (e.g., performing, visual or multidisciplinary arts) |
| Starks, 2023 | UK | n= 102 adult men at risk of/experiencing loneliness and/or social isolation -` mostly retired, widowed or living alone. | Community spaces enabling men to connect, converse, make friends, and get creative with tools and machinery, and with support from others, learn new skills. Members pay small weekly fee and take part in activities such as woodworking, metalworking, repairing and restoring furniture or bicycles, building boxes. |
| **OTHER SINGLE ACTIVITIES (N=8)** | | | |
| Blevins, 2023 | USA | Older community-dwelling adults (n not reported) | 0.5hr virtual church sessions weekly for 12 weeks consisting of opening prayer, Bible verses, mindfulness exercise, teaching, gratitude and Bible memorization |
| Chan et al., 2017 | Hong Kong | n= 45 socially isolated older adults | 3-month program of 2x 60-minute sessions each week of tai chi qigong, encouraged by a paired socially active volunteer (in and out of classes) |
| DCMS, 2019 (two intervention arms) | England | n= 20 individuals from 3 test sites: community centre users (largely with learning difficulties); supported living facility (30-60yrs) and residential home residents (60+ and living with age-related illnesses).  n= 17 individuals with care responsibilities at home | Social gaming app that brings people together to take part in online quizzing, games and chat to combat social isolation. Uses video communication to allow users to meet and participate irrespective of location, allowing users to take part in a group situation, or from their own room. Requires 5g (high bandwidth to drive device-to-device video capability).  Push to Talk device allows users to press a button, indicating that they want a chat, and be connected via their phone to another user who has also pushed their button. Users are grouped into ‘communities’ of people in similar situations. |
| Robinson et al., 2013 | New Zealand | n= 34 older adult residents in the hospital or rest home care areas of a retirement home | 12x 1hr weekly group sessions for participants to discuss and interact with Paro, an advanced interactive robot modelled after a baby seal and covered in white artificial fur. |
| Thomas et al., 2016 (two intervention arms) | USA | n= 359 older community-dwelling adults living on their own across two intervention arms | First intervention arm: daily meal delivery (traditional) by Meals on Wheels  Second intervention arm: weekly meal delivery (frozen) by Meals on Wheels |
| Tkatch et al., 2020 | USA | n= 125 older community-dwelling adults without pets | 2-months of having a weekly animatronic pet (choice of cat or dog), where participants were instructed to treat it as a pet; participants received automated weekly phone-calls reminding them to interact with their pet |
| **MULTIPLE ACTIVITIES (n=7)** | | | |
| Bartlett et al., 2012 (three intervention arms) | Australia | n= 56 older community-dwelling adults across three intervention arms | Rural town: a regular fitness programme and an arts programme was established; provisions were also made to build community capacity so participants could arrange their own activities and find funding for future development.  Coastal centre: community forums, an action plan and a buddy system for older people fostered engagement in community social welfare.  Metropolitan centre: volunteers were recruited and trained to deliver information and resources to seniors especially migrants (e.g., leisure activities and library services) |
| Basaran, 2016 | Turkey | n= 23 female adult prisoners | 1.5hr recreational sessions twice a week for 12 weeks (e.g., volleyball, karaoke, art, Zumba, orienteering) |
| Rodrıguez-Romero et al., 2021 | Spain | n= 55 older community-dwelling adults | 18x group sessions across 6 months consisting of: i) education health promotion and disease prevention sessions (e.g., healthy eating, needs of ageing, memory, sleep hygiene), ii) activities to improve mental and emotional state (e.g., mindfulness, yoga, laughter therapy, songs) and iii) activities to improve social networks and use of community resources (neighbourhood kitchen, cinema, green walk, cultural trip) |
| Roland et al., 2021 | Canada | n= 99 older community-dwelling adults | 6-months of weekday 2.5hr group-based telephone-based sessions; participants chose which and how many sessions to attend. Sessions included discussions, games, and presentations under the categories of health (e.g. disease management, exercise), recreation (e.g. BINGO, book clubs, museum talks), and education (e.g. arts and culture talks, English practice). |
| Routasalo et al., 2008 | Finland | n= 228 older community-dwelling adults | 12x weekly group sessions aimed to empower older people and promote peer support and social integration; topics were directed by the group and included art (e.g., creating art and speaking with artists), exercise (e.g., group dancing, swimming), and therapeutic writing and group therapy (e.g., writing about their experiences of loneliness and then discussing as a group). |

^a^ Sample size represents number of participants who responded to post intervention loneliness measures across intervention and control groups

**Supplementary Table S6. Characteristics of each intervention in the Social Support theme (n=46)**

| **Authors** | **Country** | **Sample size ^a^ and description** | **Intervention description** |
| --- | --- | --- | --- |
| **BEFRIENDING/MENTORING/PEER-SUPPORT (N= 20)** | | | |
| Bouwman et al., 2017 | Netherlands | n= 75 older community-dwelling adults | Online Friendship Enrichment Program; 5 online lessons to encourage participants to become aware of their social needs/desires, analyse existing social network, reflect on friendship expectations and develop new friendships |
| Burchett et al., 2022 | Wales | n= 26 older people living in supported housing and experiencing or at risk of experiencing social isolation and/or loneliness | 2 peer support groups - weekly sessions lasting ~12 weeks |
| Hernández-Ascanio, et al., 2022 | Spain | n= 94 older adults in primary care centres | 4-month CARELINK programme; 6x face-to-face home sessions (30+ min) and 5 telephone calls (20+ min) to stimulate social integration and encourage renewed socialisation |
| Juris et al., 2022 | USA | Mentees: n= 7 older community-dwelling adults  Mentors: n= 18 university students | 1-4 'reverse mentoring' sessions over a 3-month period, where student technology mentors offered solution via phone or video conferencing |
| Kahlon et al., 2021 | USA | n= 226 adults that are clients of meals on wheels | 4-weeks of receiving phone calls of <10 min (5x/week for 1st week, 2-5x/week for 3 weeks as decided by each participant); volunteers were trained in empathetic conversation techniques and told the goal was to learn something from each person they called |
| Kahlon et al., 2021 | USA | n= 226 homebound, largely single, adults who require meals from a community-based provider | Empathy-oriented (empathetic listening) telephone call program lasting 4 weeks. Initially with daily calls then dependent on need/preference. Calls lasted 10 minutes max. |
| Lai et al., 2020 | Canada | n= 60 older community-dwelling adults who have immigrated from China | 8-week peer support intervention in which volunteers (2 per participant) provide emotional support, problem-solving support, and community resource sharing through home visits and phone calls. |
| Larsen et al., 2019 (two intervention arms) | Norway | n= 1937 upper secondary school teenagers across two arms | First intervention arm: single-tier. 8-month programme in which students are trained as peer mentors and work with teachers (in group meetings throughout year) to create and maintain good psychosocial class environment.  Second intervention arm: multi-tier consisting of 1) 8-month programme in which students are trained as peer mentors and work with teachers (in group meetings throughout year) to create and maintain good psychosocial class environment and 2) a mental health support team consisting of counsellors, school nurses and follow-up services staff which targets individual students with mental health problems or those at-risk of dropping out. |
| Lorente-Martínez et al., 2021 | Spain | n= 48 older women living alone | 9x weekly 2.5hr psychosocial sessions in which volunteer university students are matched with a participant and visit them at home to talk (e.g. discuss social activities they used to enjoy or would like to try) and some sessions included visiting the local community centre together. |
| Lowthian et al., 2018 | Australia | n= 34 older patients recently discharged from emergency departments | Weekly 30min peer support phone calls from hospital volunteers to patients (beginning within 72hrs of discharge for up to 3 months, including referral for ongoing community support after this period) |
| Moonen et al., 2022 | Canada | n= 47 older community-dwelling adults living on their own | 12-weeks of regular 15-30min phone calls between volunteer medical students employing empathetic listening, and older participants during Covid-19; call timing, regularity and content was flexible lead by participants |
| Moore & Preston, 2015 | UK | n= 1669 older people at risk of loneliness and/or social isolation | Helpline and Wellbeing and Friends service that matches older people to a volunteer friend calls them for an informal chat and to provide information, advice, referrals to other organisations and an informal chat. Survey administered to users of Wellbeing and Friends service who receive calls minimum once/week of varying lengths (time not specified) |
| Ramamonjiarivelo et al., 2022 | USA | n= 22 older community-dwelling adults | 6x 30-min weekly 1:1 virtual interaction (via phone, text, or video call) as part of an intergenerational virtual university led service-learning between undergraduate students and older adults |
| Renaisi, 2016 | England | n= 35 older people and young people at the start of their professional lives - both groups at risk of loneliness and/or social isolation | Programmes run in North and South London to improve community networks of young professionals and older neighbours. Projects include creating 'Love Your Neighbour' 1:1 friendships, social clubs, 'Winter Wellbeing' outreach and community fundraising. |
| Roberts and Windle, 2019 | Wales | n= 114 older community-dwelling adults | 10-15x weekly meetings between volunteers paired with older to offer companionship, practical and psychological support, and to increase social networks, confidence and independence |
| Sandu et al., 2021 | USA | n= 84 older community dwelling adults | 1-year of ~10min weekly phone calls from a student volunteer to an older adult, following standardized call scripts |
| Simpson, 2014 | England | n= 46 adults diagnosed with mental illness discharged from mental health ward | 6-weeks of peer support beginning 2-3 weeks before discharge and continuing 4-weeks after |
| Stewart et al., 2011 | Canada | n= 58 adult Somali and Sudanese refugees | 6x biweekly 1-1.5hr in-person support groups (5-12 people), with 1:1 support given to new refugees with additional 1:1 support via the phone between sessions. Participants directed topics of discussion around enhancing cultural understanding and social integration (e.g. promoting new skills, seeking employment, improving family dynamics, and overcoming discrimination). Peer facilitators were Sudanese and Somali people who had been settled in Canada for 10+ years |
| Theurer et al., 2021a | Canada | Mentees: n= 43 older adult care home residents  Mentors: n=27 older adult care home residents | 6-months of weekly 2hr activities at long-term care homes consisting of: i) team meetings with activities and short educational sessions for residents and mentors (26 modules learning communication skills and about topics such as “What if They Don’t Talk Much,” and “Supporting People Grieving.”); b) visits between mentors and mentees to provide empathetic support and encouragement for mentees to attend community events. |
| **EDUCATIONAL/SOCIAL SKILLS DEVELOPMENT (N= 6)** | | | |
| Bostick and Anderson, 2009 | USA | n= 49 children with social skills deficits | Social Skills Group Intervention; 10 group sessions (duration nr) focusing on development of verbal and nonverbal communication, initiation, cooperation, compromise and negotiation skills |
| Coll-Planas et al., 2017 | Spain | n= 26 older community-dwelling adults | 15x 1.5hr weekly group-based sessions discussing loneliness, social participation, community assets and engaging in the community |
| Costello et al., 2022 | USA | n= 438 recent first-year and transfer students at a public university | The Connection Project; 9x 60‐ to75‐minute weekly sessions consisting of activities and discussions to teach and develop deep, supportive, and authentic relationships |
| Haslam et al., 2019 | Australia | n= 99 adults with symptoms of depression | Groups 4 Health; 4x weekly 1-1.5hr group sessions working through activities and exercises to develop group connection (e.g., social awareness, social mapping, maximising, building and sustaining relationships), followed by a final session 4 weeks later, |
| Lim et al., 2020 | Australia | n= 10 young people with psychosis | 3-months of a gamified app (i.e., with points, challenges etc) aimed to develop positive interpersonal skills (e.g., showing kindness and reciprocity within relationships) through tasks delivered daily via videos and audio. |
| Mattanah et al., 2010 | USA | n= 112 first-year university students | 9x 1.5hr group social support discussions spread across school year; sessions were facilitated by two undergraduate clinical psychology students and semi-structured around topics such as maintaining old friendships, etc. |
| **SOCIAL PRESCRIBING/CONNECTOR SERVICE (N= 6)** | | | |
| Foster et al., 2020 | England | n= 2250 adults experiencing loneliness | Social prescribing service with up to 12 weeks support from a link worker helping them connect with social groups, session duration unreported |
| Kellezi et al., 2019 | England | n= 19 adults with chronic health conditions (not specified) and/or at-risk of loneliness | 8x weekly support sessions, where health coaches/link worker provide patients with practical and emotional support and patients are supported to join third-sector groups (e.g., voluntary or social enterprise) |
| Llewellyn et al., 2020 | Wales | n= 7 adults experiencing mild/moderate mental health problems and/or emotional wellbeing disorders | Link worker SP service with several clinical, third sector routes and self-referral to Mind link worker. Participants included in the trial only came from GP or another primary care worker within the practice; Initial needs assessment followed by co-creation of plan to access community services and support. |
| Massie & Ahmad, 2019 | England | n= 51 people with non-clinical needs, referred to the social prescribing service by GPs, practice nurses, community nursing teams, care navigation staff, social workers, ambulance staff and A&E staff | 3 x link workers placed across the city to help individuals access range of health and wellbeing services. Support commonly included exercise programmes, social activities, healthy eating, self-management of long-term conditions and support with welfare benefits and financial issues. Includes one face-to-face meeting then follow-up meetings depending on progress. |
| McDaid et al., 2021 | England | n= 121 older people experiencing or at risk of experiencing social isolation and/or loneliness | Personalised support delivered over 6-9 months. Volunteer matching or case worker develops personalised plan and then refers to appropriate emotional and practical support aimed at rebuilding confidence and support connections with people, places, or activities in their community. |
| Red Cross & The Coop Foundation, 2019 | UK | Individuals experiencing or at risk of experiencing social isolation and/or loneliness; half of support recipients undergoing life transitions (largely due to physical & mental health issues and mobility limitations) | Community Connector service providing person-centered support to re-connect people feeling lonely or socially isolated back to their communities. Signposting to local groups and activities, including for emotional and practical support. Typically involved 8 support sessions for up to 3 months. |
| **ICT training/ Equipment provision (N= 5)** | | | |
| ﻿Antunes et al., 2022 | Brazil | n= 36 older adults enrolled in computer classes for beginners at an ageing care centre | 2hr sessions twice a week for 4 weeks (8 computer science classes), followed by the same format programme but with 15 mins per class dedicated to digital games (16 classes total); participants arranged into six groups with maximum group size of 10 |
| Fields J. et al., 2021 | USA | n= 57 socially isolated older adults | 8x weekly 1-to-1 in-home lessons delivered by a volunteer covering how to use an iPad, email, applications, online communities, online safety and online fun |
| Gadbois et al., 2022 | USA | n= 18 homebound older adults | 1.5-2hr 1-to-1 technology training sessions per week for 4 weeks then 30 mins phone assistance per week for 10 weeks |
| Mullins et al., 2020 | USA | n= 11 older adults living in US Housing and Urban Development | 3x computer classes (basic computer skills, internet safety/email/social media, computer Q&A) each offered at 8 different times for residents |
| Quinn, 2021 | USA | n= 36 older community-dwelling adults | 4x 2hr weekly social media group training sessions held in a classroom (75% Facebook, 25% Twitter focusing on online security, online etiquette, messaging and photo sharing) |
| **OTHER (N= 9)** | | | |
| Broer et al., 2011 | Netherlands | n= 186 intellectually disabled adults | 29 teams of healthcare providers, each targeting a group of 20-30 intellectually disabled or psychiatric patients; teams and clients worked together to create individual plans to increase social networks |
| Jones et al., 2021 | USA | n= 16 older community-dwelling adults living on their own | Participants used an Amazon Echo personal voice assistant for 4-weeks (min 5 interactions/ day), then as much as they liked for a further 4-weeks. |
| Kramer et al., 2022 | Netherlands | n= 32 older community-dwelling adults living on their own | 8-week access to an eHealth app which uses AI conversational agents to provide motivation for decreasing loneliness and improving eating behaviour |
| Larsson et al., 2016 | Sweden | n= 28 older community-dwelling adults | 4-month multi-tier intervention consisting of 1) 1.5hr biweekly educational group meetings and 2)1.5hr weekly individual meetings with an occupational therapist. Aim of meetings was to assist participants with goal-orientated social internet-based activities and attain goals (e.g., make a new friend or learn to participate in debates) |
| Nguyen et al., 2022 | USA | n= 124 adult caregivers of people with dementia | Participants were given a tablet with software designed for older adults to support cognitive, emotional, social, physical, and spiritual wellness (games, quizzes, tv shows, audiobooks, music, etc).  Participants received an initial group 1hr online training session, met monthly in a 1hr online support group with other participants, and received digital delivery of a newsletter (weekly for first 6-months, monthly for the last 6-months) highlighting a different tablet feature and reminding participants about local support groups. |
| Ohta et al., 2022 | Japan | n= 77 community-dwelling adults | 16x meetings (i.e. Osekkai conferences) held at care centres to discuss community problems and organise voluntary resolutions held across a one-year period; Osekkai is a traditional Japanese behaviour in which people do what they think is good for others, which can increase people’s social participation. |
| Taube et al., 2018 | Sweden | n= 108 older community-dwelling adults receiving care | Out-patient case management intervention involved each patient having two case managers (a nurse and a physiotherapist) which made monthly visits (sometimes together), or more as required, over 12-months. Case managers were available to call for assistance at any time, and shared information with participants about the consequences of ageing and local activities as well as individually tailored information regarding disease and recovery management. |
| The Mental Health Foundation, 2018 | UK | n= 13 older people living in support housing | Self-management initiative with peer support groups. Weekly sessions for 6 months facilitate and provide peer support to people living in housing schemes. Included activities designed to increase meaningful participation and aid cognitive stimulation, focusing on the participants’ identity and their passions |
| Traverse, 2018 | UK | n= 14 home sharers - older people and others who need support to stay in their homes (e.g. divorcees, long-distance commuters); homeholders - young professionals | Homeshare scheme in eight pilot sites allowing two people to share a home for mutual benefit. More commonly brings together older people with young people who provide companionship and 10 hr per/week of low-level practical support in return for an affordable place to live. |

^a^ Sample size represents number of participants who responded to post intervention loneliness measures across intervention and control groups

**Supplementary Table S7. Characteristics of each intervention in ‘Multiple themes’ (n=9)**

| **Authors** | **Country** | **Sample size ^a^ and description** | **Intervention description** |
| --- | --- | --- | --- |
| Honigh-de Vlaming et al., 2013 | Netherlands | n= 858 older community-dwelling adults | Five intervention components: a mass media campaign, information meetings for local elderly people, psychosocial group courses for persons with mental health problems or chronic diseases, social activation by the community-based Neighbours Connected intervention, and training of intermediaries (homecare nurses, municipal advisors, and volunteers) |
| Pynnönen et al., 2017 | Finland | n= 222 older community-dwelling adults | 6-month social intervention of choice (weekly group supervised exercise, weekly group social activity, or personal counselling every 3rd week) vs. self-directed control group (single counselling session). |
| Dayson et al., 2021 | England | n= 608 older people at risk of loneliness and/or social isolation | Group-based and one-to-one therapeutic and befriending activities based on the ‘Five Ways to Wellbeing'. Evaluation cohort attended activities that included practical advice and guidance, and social activities. |
| Leyland et al., 2022 | England | n= 771 older people at risk of loneliness and/or social isolation | Part of BL Ageing Better programme (Citywide-Leeds). 19 projects included, all worked with local community, co-produced projects, recruited volunteers, and sometimes, linked with other organisations. Projects include community-based development approaches. Evaluation cohort activities include: meal-sharing activities; cultural appropriate group activities; walking/outdoor interventions & arts/theatre/dance activities. |
| Moreton et al., 2019 | England | n= 450 older people at risk of loneliness and/or social isolation | Part of wider Ageing Better programme - Voluntary sector-led partnerships in 14 areas across England aimed at promoting community action on ageing and isolation. City-wide programme composed of networks providing a range of group activities: including Exercise (42 Networks), Arts, Food-related, trips (27 Networks) and skills workshops (24 Networks). |
| Ecorys, 2021 | England | n= 1498 older people experiencing or at risk of experiencing social isolation and/or loneliness | Ageing better. Strategic programme delivered by 14 VCSE sector-led partnerships. Develops/delivers plans to create new and enjoyable ways for people over 50 to be actively involved in their communities to combat social isolation and loneliness. Intervention types include ICT intervention Asset-based community development (ABCD), Creative activities, Social interventions, Culture change, Knowledge sharing/building, Social prescribing, Mental health, Physical health and Transport. |
| Jones et al., 2021 | England | n= 897 older people experiencing or at risk of experiencing social isolation and/or loneliness | Part of wider Ageing Better programme - voluntary sector-led partnerships in 14 areas across England aimed at promoting community action on ageing and isolation. City-wide programme included projects providing 121 and group support: Intergenerational Activity, Creativity and Arts, Peer Support, Food and Nutrition and Talking therapies. |
| Rose et al., 2021 | England | n= 0 individuals across the lifespan experiencing or at risk of experiencing social isolation and/or loneliness | Cross-govt fund that gave 126 VCSE sector orgs grants to deliver 121 & group service delivery, as well as system-level campaigning/change. Cohort of 23 grant holders whose activities largely focused on: Befriending/peer-to-peer support, advice, signposting & outreach, Group activities - arts and leisure, educational approaches, physical leisure activities and therapeutic support. Average engagement in cohort was 1-9 hours p/m for 3-12 months p/y. |
| Martin et al., 2021 | England | n= 2171 adults at risk of loneliness during covid-19 restrictions; multiple groups of vulnerable adults and their families (vulnerabilities include: physical & mental health-related, carer-status-related, financial difficulties, immigration-related) new mothers; adults at risk of loneliness during covid-19 restrictions) | Fund aimed at supporting delivery of services aimed at addressing loneliness and to help organisations adapt services during Covid-19 restrictions. Evaluation cohort composed of 8 VCSE sector orgs delivering following primary types of activity: Befriending, technological support (eg. Using ICT equipment) |

^a^ Sample size represents number of participants who responded to post intervention loneliness measures across intervention and control groups

**Supplementary Figure S1. Funnel plot to assess publication bias in included studies from a) pre- and post-intervention changes in intervention group and b) differences in pre- and post-intervention loneliness scores between control and intervention groups.**

**Supplementary Figure S2. Subgroup meta-analysis of age differences in social interaction interventions (n=15 studies)**


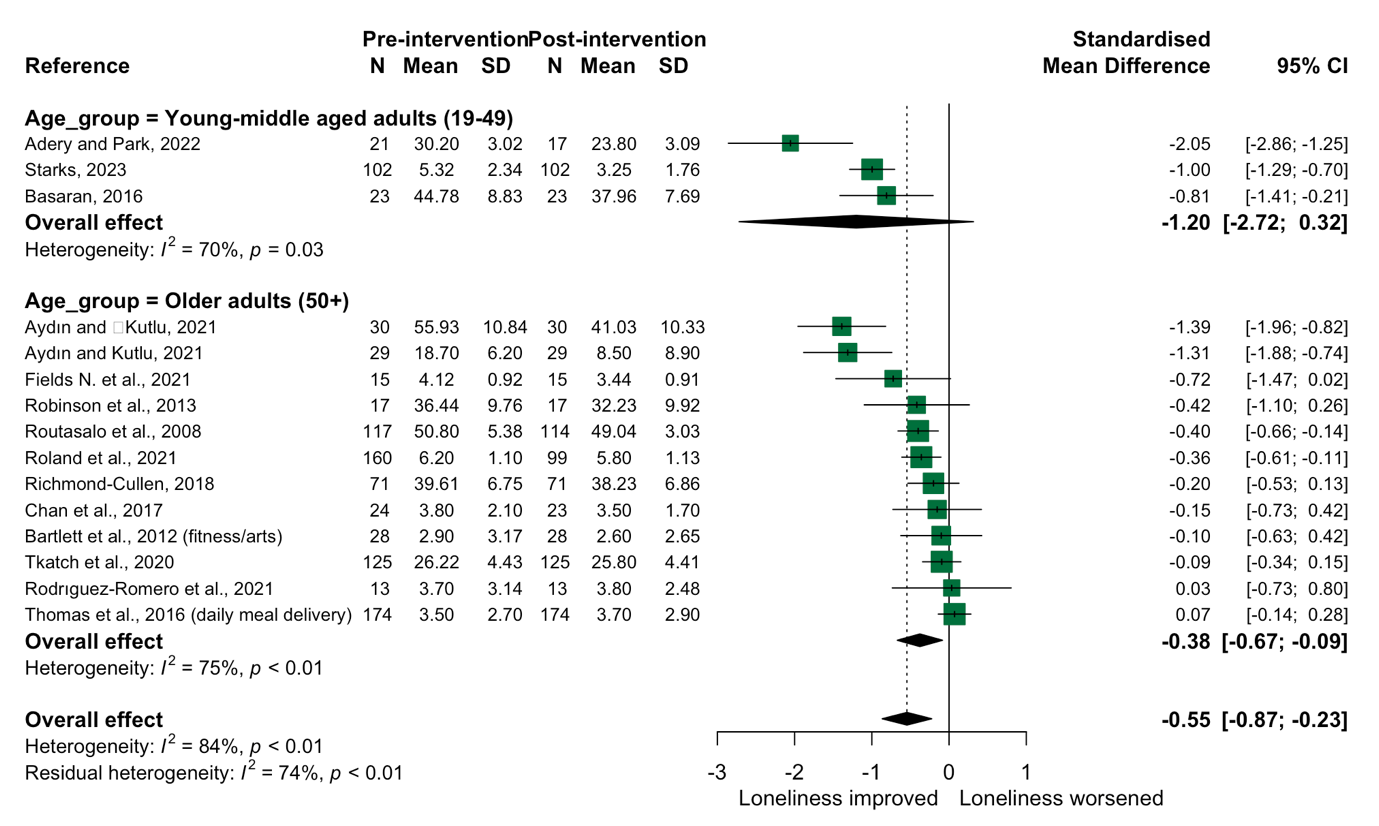


**Supplementary Text S2. Reference list of studies included in review**

Adery, L. H., & Park, S. (2022). A pilot choral intervention in individuals with schizophrenia-spectrum conditions; Singing away loneliness. *PsyCh journal*, *11*(2), 227-231.

Antunes, T. P. C., de Mello Monteiro, C. B., eira, Crocetta, T. B., de Lima Antao, J. Y. F., Leitao, F. N. C., . . . de Abreu, L. C. (2022). Digital games in the computer classes to reduce loneliness of individuals during aging. *Current Psychology: A Journal for Diverse Perspectives on Diverse Psychological Issues*, No-Specified.

Apteligen Ltd. (2021). *Evaluation of the Carers’ Music Fund: Final report* <https://spiritof2012.org.uk/wp-content/uploads/2021/12/2021-06-23-Carers-Music-Fund-evaluation-1.pdf>

Aydin, M., & Kutlu, F. Y. (2021). The Effect of Group Art Therapy on Loneliness and Hopelessness Levels of Older Adults Living Alone: A Randomized Controlled Study. *Florence Nightingale journal of nursing*, *29*(3), 271-284.

Bartlett, H., Warburton, J., Lui, C.-W., Peach, L., & Carroll, M. (2013). Preventing social isolation in later life: Findings and insights from a pilot Queensland intervention study. *Ageing & Society*, *33*(7), 1167-1189.

Basaran, Z. (2016). The Effect of Recreational Activities on the Self-Esteem and Loneliness Level of the Prisoners as an Alternative Education. *Universal Journal of Educational Research*, *4*(5), 1080-1088.

Blevins, D. (2023). A Faith-Based Intervention to Address Social Isolation and Loneliness in Older Adults. *Journal of Christian nursing : a quarterly publication of Nurses Christian Fellowship*, *40*(1), 28-35.

Bostick, D., & Anderson, R. (2009). Evaluating a small-group counseling program-A model for program planning and improvement in the elementary setting. *Special Issue: Action research in school counseling.*, *12*(6), 428-433.

Bouwman, T. E., Aartsen, M. J., van Tilburg, T. G., & Stevens, N. L. (2017). Does stimulating various coping strategies alleviate loneliness? Results from an online friendship enrichment program. *Journal of social and personal relationships*, *34*(6), 793-811.

Broer, T., Nieboer, A. P., Strating, M. M. H., Michon, H. W. C., & Bal, R. A. (2011). Constructing the social: An evaluation study of the outcomes and processes of a 'social participation' improvement project. *Journal of Psychiatric and Mental Health Nursing*, *18*(4), 323-332.

Brown, J., Brown, K., Clayton, D., Mcgill, G., Padley, W., & Vries, K. D. (2019). *Local History Cafes: An Evaluation of the Initial Programme. Museum Development East Midlands*

Bruehlman-Senecal, E., Hook, C. J., Pfeifer, J. H., Fitzgerald, C., Davis, B., Delucchi, K. L., . . . Ramo, D. E. (2020). Smartphone App to Address Loneliness Among College Students: Pilot Randomized Controlled Trial. *JMIR Mental Health*, *7*(10), e21496.

Burchett, N., Elford, R., & Robinson, E. (2022). *Standing Together Cymru*.

Caputi, M., Cugnata, F., & Brombin, C. (2021). Theory of mind and loneliness: Effects of a conversation-based training at school. *International journal of psychology : Journal international de psychologie*, *56*(2), 257-265.

Chan, A. W., Yu, D. S., & Choi, K. C. (2017). Effects of tai chi qigong on psychosocial well-being among hidden elderly, using elderly neighborhood volunteer approach: a pilot randomized controlled trial. *Clinical interventions in aging*, *12*, 85-96.

Cohen-Mansfield, J., Hazan, H., Lerman, Y., Shalom, V., Birkenfeld, S., & Cohen, R. (2018). Efficacy of the I-SOCIAL intervention for loneliness in old age: Lessons from a randomized controlled trial. *Journal of psychiatric research*, *99*, 69-75.

Coll-Planas, L., Del Valle Gomez, G., Bonilla, P., Masat, T., Puig, T., & Monteserin, R. (2017). Promoting social capital to alleviate loneliness and improve health among older people in Spain. *Health & social care in the community*, *25*(1), 145-157.

Costello, M. A., Nagel, A. G., Hunt, G. L., Rivens, A. J., Hazelwood, O. A., Pettit, C., & Allen, J. P. (2022). Facilitating connection to enhance college student well-being: Evaluation of an experiential group program. *American journal of community psychology*, *70*(3), 314-326.

Creswell, J. D., Irwin, M. R., Burklund, L. J., Lieberman, M. D., Arevalo, J. M. G., Ma, J., . . . Cole, S. W. (2012). Mindfulness-Based Stress Reduction training reduces loneliness and pro-inflammatory gene expression in older adults: a small randomized controlled trial. *Brain, behavior, and immunity*, *26*(7), 1095-1101.

Cruwys, T., Haslam, C., Rathbone, J. A., Williams, E., Haslam, S. A., & Walter, Z. C. (2022). Groups 4 Health versus cognitive-behavioural therapy for depression and loneliness in young people: randomised phase 3 non-inferiority trial with 12-month follow-up. *The British journal of psychiatry : the journal of mental science*, *220*(3), 140-147.

Dayson, C., Harris, C., Bashir, N., Bennett, E., & Woodward, A. (2022). *Evaluation of Age Better in Sheffield; The Impact on Social Isolation and Loneliness (2015-21)*.

DCMS, & Liverpool 5g Consortium. (2019 ). *Liverpool 5G Health and Social Care Testbed: Benefits, Outcomes and Impact*. <https://liverpool5g.org.uk/wp-content/uploads/2020/04/Liverpool-5G-Testbed-Benefits-Outcomes-Impact.pdf>

Ecorys, Campbell-Jack, D., Humphreys, A., Whitley, J., Williams, J., & Cox, K. (2021). *Ageing Better: Impact Evaluation Report.* .

Fields, J., Cemballi, A. G., Michalec, C., Uchida, D., Griffiths, K., Cardes, H., . . . Lyles, C. R. (2021). In-Home Technology Training Among Socially Isolated Older Adults: Findings From the Tech Allies Program. *Journal of applied gerontology : the official journal of the Southern Gerontological Society*, *40*(5), 489-499.

Fields, N., Xu, L., Greer, J., & Murphy, E. (2021). Shall I compare thee...to a robot? An exploratory pilot study using participatory arts and social robotics to improve psychological well-being in later life. *Aging & mental health*, *25*(3), 575-584.

Foster, A., Thompson, J., Holding, E., Ariss, S., Mukuria, C., Jacques, R., . . . Haywood, A. (2021). Impact of social prescribing to address loneliness: A mixed methods evaluation of a national social prescribing programme. *Health & social care in the community*, *29*(5), 1439-1449.

Gadbois, E. A., Jimenez, F., Brazier, J. F., Davoodi, N. M., Nunn, A. S., Mills, W. L., . . . Thomas, K. S. (2022). Findings From Talking Tech: A Technology Training Pilot Intervention to Reduce Loneliness and Social Isolation Among Homebound Older Adults. *Innovation in aging*, *6*(5), igac040.

Gurgan, U. (2013). The Effect of Psychological Counselling in Group on Life Orientation and Loneliness Levels of the University Students. *Educational Research and Reviews*, *8*(24), 2303-2312.

Hansen, P., Main, C., & Hartling, L. (2021). Dance Intervention Affects Social Connections and Body Appreciation Among Older Adults in the Long Term Despite COVID-19 Social Isolation: A Mixed Methods Pilot Study. *Frontiers in psychology*, *12*, 635938.

Haslam, C., Cruwys, T., Chang, M. X. L., Bentley, S. V., Haslam, S. A., Dingle, G. A., & Jetten, J. (2019). GROUPS 4 HEALTH reduces loneliness and social anxiety in adults with psychological distress: Findings from a randomized controlled trial. *Journal of consulting and clinical psychology*, *87*(9), 787-801.

Hernandez-Ascanio, J., Perula-de Torres, L. A., Rich-Ruiz, M., Gonzalez-Santos, J., Mielgo-Ayuso, J., Gonzalez-Bernal, J., & Group, A. S. S. C. (2023). Effectiveness of a multicomponent intervention to reduce social isolation and loneliness in community-dwelling elders: A randomized clinical trial. *Nursing open*, *10*(1), 48-60.

Honigh-de Vlaming, R., Haveman-Nies, A., Heinrich, J., van't Veer, P., & de Groot, L. C. P. G. M. (2013). Effect evaluation of a two-year complex intervention to reduce loneliness in non-institutionalised elderly Dutch people. *BMC public health*, *13*, 984.

Iyer, R. B., Vadlapudi, S., Iyer, L., Kumar, V., Iyer, L., Sriram, P., . . . Iyer, P. (2022). Impact of the Heartfulness program on loneliness in high schoolers: Randomized survey study. *Applied psychology. Health and well-being*.

Jang, M., & Kim, Y. (2012). The effect of group sandplay therapy on the social anxiety, loneliness and self-expression of migrant women in international marriages in South Korea. *The Arts in Psychotherapy*, *39*(1), 38-41.

Jones, M., Beardmore, A., & White, J. (March 2021). *Effects of Bristol Ageing Better Projects for Older People: Evaluation of the impacts of the programme on loneliness, isolation and a range of associated outcomes*. <https://uwe-repository.worktribe.com/output/7490342>

Jones, V. K., Hanus, M., Yan, C., Shade, M. Y., Blaskewicz Boron, J., & Maschieri Bicudo, R. (2021). Reducing Loneliness Among Aging Adults: The Roles of Personal Voice Assistants and Anthropomorphic Interactions. *Frontiers in public health*, *9*, 750736.

Juris, J. J., Bouldin, E. D., Uva, K., Cardwell, C. D., Schulhoff, A., & Hiegl, N. (2022). Virtual Intergenerational Reverse-Mentoring Program Reduces Loneliness among Older Adults: Results from a Pilot Evaluation. *International journal of environmental research and public health*, *19*(12).

Kahlon, M. K., Aksan, N., Aubrey, R., Clark, N., Cowley-Morillo, M., Jacobs, E. A., . . . Tomlinson, S. (2021). Effect of Layperson-Delivered, Empathy-Focused Program of Telephone Calls on Loneliness, Depression, and Anxiety Among Adults During the COVID-19 Pandemic: A Randomized Clinical Trial. *JAMA psychiatry*, *78*(6), 616-622.

Kall, A., Back, M., Welin, C., Aman, H., Bjerker, R., Wanman, M., . . . Andersson, G. (2021). Therapist-Guided Internet-Based Treatments for Loneliness: A Randomized Controlled Three-Arm Trial Comparing Cognitive Behavioral Therapy and Interpersonal Psychotherapy. *Psychotherapy and psychosomatics*, *90*(5), 351-358.

Kall, A., Jagholm, S., Hesser, H., Andersson, F., Mathaldi, A., Norkvist, B. T., . . . Andersson, G. (2020). Internet-Based Cognitive Behavior Therapy for Loneliness: A Pilot Randomized Controlled Trial. *Behavior therapy*, *51*(1), 54-68.

Kellezi, B., Wakefield, J. R. H., Stevenson, C., McNamara, N., Mair, E., Bowe, M., . . . Halder, M. M. (2019). The social cure of social prescribing: a mixed-methods study on the benefits of social connectedness on quality and effectiveness of care provision. *BMJ Open*, *9*(11), e033137.

Kramer, L. L., van Velsen, L., Clark, J. L., Mulder, B. C., & de Vet, E. (2022). Use and Effect of Embodied Conversational Agents for Improving Eating Behavior and Decreasing Loneliness Among Community-Dwelling Older Adults: Randomized Controlled Trial. *JMIR formative research*, *6*(4), e33974.

Lai, D. W. L., Li, J., Ou, X., & Li, C. Y. P. (2020). Effectiveness of a peer-based intervention on loneliness and social isolation of older Chinese immigrants in Canada: a randomized controlled trial. *BMC geriatrics*, *20*(1), 356.

Larsen, T. B., Urke, H., Tobro, M., Ardal, E., Waldahl, R. H., Djupedal, I., & Holsen, I. (2021). Promoting mental health and preventing loneliness in upper secondary school in Norway: Effects of a randomized controlled trial. *Scandinavian Journal of Educational Research*, *65*(2), 181-194.

Larsson, E., Padyab, M., Larsson-Lund, M., & Nilsson, I. (2016). Effects of a social internet-based intervention programme for older adults: An explorative randomised crossover study. *The British Journal of Occupational Therapy*, *79*(10), 629-636.

Leyland, A., Naughton-Doe, R., Wigfield, A., & Martin, C. (2022). *Time to Shine local evaluation, Report 6: participation, engagement and outcomes for older people* <https://www.opforum.org.uk/wp-content/uploads/2022/07/Report-6_TTS-Local-evaluation_June-2022.pdf>

Lim, M. H., Gleeson, J. F. M., Rodebaugh, T. L., Eres, R., Long, K. M., Casey, K., . . . Penn, D. L. (2020). A pilot digital intervention targeting loneliness in young people with psychosis. *Social psychiatry and psychiatric epidemiology*, *55*(7), 877-889.

Llewellyn, M., Wallace, S., Wallace, C., Elliott, M., Williams, M., & Ganesh, S. (December 2020). *Evaluation of the pilot mental health social prescribing programme, Final Report for Mind Cymru*. <https://www.mind.org.uk/media/7603/mind-social-prescribing-final-report-141220-whisc.pdf>

Lorente-Martinez, R., Brotons-Rodes, P., & Sitges-Macia, E. (2022). Benefits of a psychosocial intervention programme using volunteers for the prevention of loneliness among older women living alone in Spain. *Health & social care in the community*, *30*(5), 2000-2012.

Lowthian, J. A., Lennox, A., Curtis, A., Wilson, G., Rosewarne, C., Smit, D. V., . . . Dale, J. (2018). HOspitals and patients WoRking in Unity (HOW R U?): telephone peer support to improve older patients' quality of life after emergency department discharge in Melbourne, Australia-a multicentre prospective feasibility study. *BMJ Open*, *8*(6), e020321.

Macmillan, T., Ronca, M., Bidey, T., & Rembiszewsk, P. (March 2018 ). *Evaluation of the Homeshare pilots, Final report*. <https://www.housinglin.org.uk/_assets/Resources/Housing/OtherOrganisation/Homeshare-Evaluation-Report.pdf>

Martin, A., Crowley, J., Pinto, C., Shipsey, F., Martin, I., Windle, K., & Dinos, S. (2021). *Covid-19 Loneliness Fund: A process evaluation*. <https://assets.publishing.service.gov.uk/government/uploads/system/uploads/attachment_data/file/1010052/Covid-19_Loneliness_Fund_Evaluation__accessible_version_.pdf>

Massie, R., & Ahmad, N. (2019 ). *An evaluation of Wolverhampton’s Social Prescribing Service: A New Route to Wellbeing*.

Mattanah, J. F., Ayers, J. F., Brand, B. L., Brooks, L. J., Quimby, J. L., & McNary, S. W. (2010). A social support intervention to ease the college transition: Exploring main effects and moderators. *Journal of College Student Development*, *51*(1), 93-108.

McDaid, D., Park, A., & Fernandez, J. (2021). *Reconnections: Impact evaluation report* <https://www.lse.ac.uk/cpec/assets/documents/Reconnections.pdf>

Moonen, G., Perrier, L., Meiyappan, S., Akhtar, S., & Crampton, N. (2022). COVID-19 pandemic partnership between medical students and isolated elders improves student understanding of older adults' lived experience. *BMC geriatrics*, *22*(1), 636.

Moore, S., & Preston, C. (2015). *The Silver Line: Tackling Loneliness in Older People*.

Moreton, R., Stutz, A., Richards, S., Choudhoury, A., & Daly, G. (2019). *Evaluation of Ageing Better in Birmingham - Year 2 report* <https://www.bvsc.org/Handlers/Download.ashx?IDMF=baa5c034-f8eb-4dc2-a748-d22e439cbc0a>

Mueller, N. E., & Cougle, J. R. (2023). Building Closer Friendships in social anxiety disorder: A randomized control trial of an internet-based intervention. *Journal of behavior therapy and experimental psychiatry*, *78*, 101799.

Mullins, L. B., Skemp, L., Reed, D., & Emerson, M. (2020). Internet Programming to Reduce Loneliness and Social Isolation in Aging. *Research in gerontological nursing*, *13*(5), 233-242.

Nguyen, L. T., Prophater, L. E., Fazio, S., Hulur, G., Tate, R., Sherwin, K., . . . Haley, W. E. (2022). Project VITAL at Home: Impact of Technology on Loneliness and Well-Being of Family Caregivers of People with Dementia. *Clinical gerontologist*, 1-12.

Ohta, R., Maiguma, K., Yata, A., & Sano, C. (2022). A Solution for Loneliness in Rural Populations: The Effects of Osekkai Conferences during the COVID-19 Pandemic. *International journal of environmental research and public health*, *19*(9).

Ozturk, F. O., & Tekkas-Kerman, K. (2022). The effect of online laughter therapy on depression, anxiety, stress, and loneliness among nursing students during the Covid-19 pandemic. *Archives of psychiatric nursing*, *41*, 271-276.

Pynnonen, K., Rantanen, T., Kokko, K., Tiikkainen, P., Kallinen, M., & Tormakangas, T. (2018). Associations between the dimensions of perceived togetherness, loneliness, and depressive symptoms among older Finnish people. *Aging & mental health*, *22*(10), 1329-1337.

Quinn, K. (2021). Social media and social wellbeing in later life. *Special Issue: New frontiers in international retirement migration*, *41*(6), 1349-1370.

Ramamonjiarivelo, Z., Osborne, R., Renick, O., & Sen, K. (2022). Assessing the Effectiveness of Intergenerational Virtual Service-Learning Intervention on Loneliness and Ageism: A Pre-Post Study. *Healthcare (Basel, Switzerland)*, *10*(5).

Red Cross & The Coop Foundation. (2019 ). *Tackling Loneliness and Isolation: Findings from the evaluation of our Connecting Communities service*.

Renaisi. (2016). *North and South London Cares: Evaluation and Development through the Centre for Social Action Innovation Fund*. <https://media.nesta.org.uk/documents/north_and_south_london_cares_-_final_report_csaif_evaluation_support.pdf>

Richmond-Cullen, C. (2018). The effect of an artist in residence program on self-reported loneliness in senior citizens. *Educational Gerontology*, *44*(7), 425-432.

Roberts, J. R., & Windle, G. (2020). Evaluation of an intervention targeting loneliness and isolation for older people in North Wales. *Perspectives in public health*, *140*(3), 153-161.

Robinson, H., Macdonald, B., Kerse, N., & Broadbent, E. (2013). The psychosocial effects of a companion robot: a randomized controlled trial. *Journal of the American Medical Directors Association*, *14*(9), 661-667.

Rodriguez-Romero, R., Herranz-Rodriguez, C., Kostov, B., Gene-Badia, J., & Siso-Almirall, A. (2021). Intervention to reduce perceived loneliness in community-dwelling older people. *Scandinavian journal of caring sciences*, *35*(2), 366-374.

Roland, H., Ilin Shpilkerman, Y., Schaub, J., & Comeau, A.-C. (2021). Connection Through Calls: The Impact of a Seniors Center Without Walls on Older Adults' Social Isolation and Loneliness. *Gerontology & geriatric medicine*, *7*.

Rose, A., Abrams, T., Parker, E., & Todres, G. (2021). *The Building Connections Fund Part one: Evaluation of the Building Connections Fund prior to the Covid-19 pandemic*. <https://assets.publishing.service.gov.uk/government/uploads/system/uploads/attachment_data/file/1010470/BCF_Part_1_evaluation_report_May_2021.pdf>

Routasalo, P. E., Tilvis, R. S., Kautiainen, H., & Pitkala, K. H. (2009). Effects of psychosocial group rehabilitation on social functioning, loneliness and well-being of lonely, older people: randomized controlled trial. *Journal of advanced nursing*, *65*(2), 297-305.

Sandu, S., Sreedhar, S., Chang, L., Cohen, L., Cruz, A., Olson, H. R., . . . Carrion, A. (2021). 21st Century Good Neighbor Program: An Easily Generalizable Program to Reduce Social Isolation in Older Adults. *Frontiers in public health*, *9*, 766706.

Shapira, S., Cohn-Schwartz, E., Yeshua-Katz, D., Aharonson-Daniel, L., Clarfield, A. M., & Sarid, O. (2021). Teaching and Practicing Cognitive-Behavioral and Mindfulness Skills in a Web-Based Platform among Older Adults through the COVID-19 Pandemic: A Pilot Randomized Controlled Trial. *International journal of environmental research and public health*, *18*(20).

Simpson, A., Flood, C., Rowe, J., Quigley, J., Henry, S., Hall, C., . . . Bowers, L. (2014). Results of a pilot randomised controlled trial to measure the clinical and cost effectiveness of peer support in increasing hope and quality of life in mental health patients discharged from hospital in the UK. *BMC psychiatry*, *14*, 30.

Stacey, J., & Edwards, A. (2013). Resisting loneliness' dark pit: a narrative therapy approach. *Tizard Learning Disability Review*, *18*(1), 20-27.

Starks, L. (2023). *Researching the benefits and impact of Men's Sheds on their members* UK Men's Sheds Association

Stewart, L. A., Dispenza, F., Parker, L., Chang, C. Y., & Cunnien, T. (2014). A pilot study assessing the effectiveness of an animal-assisted outreach program. *Journal of Creativity in Mental Health*, *9*(3), 332-345.

Stewart, M., Simich, L., Beiser, M., Makumbe, K., Makwarimba, E., & Shizha, E. (2011). Impacts of a social support intervention for Somali and Sudanese refugees in Canada. *Ethnicity and Inequalities in Health and Social Care*, *4*(4), 186-199.

Tatlilioglu, K. (2013). The Effect of Cognitive Behavioral Oriented Psycho Education Program on Dealing with Loneliness: An Online Psychological Counseling Approach. *Education*, *134*(1), 101-109.

Taube, E., Kristensson, J., Midlov, P., & Jakobsson, U. (2018). The use of case management for community-dwelling older people: the effects on loneliness, symptoms of depression and life satisfaction in a randomised controlled trial. *Scandinavian journal of caring sciences*, *32*(2), 889-901.

The Mental Health Foundation. (2018). *An Evaluation of the Standing Together project* <https://www.mentalhealth.org.uk/sites/default/files/2022-09/standing-together-evaluation-WEB.pdf>

Theeke, L. A., Mallow, J. A., Moore, J., McBurney, A., Rellick, S., & VanGilder, R. (2016). Effectiveness of LISTEN on loneliness, neuroimmunological stress response, psychosocial functioning, quality of life, and physical health measures of chronic illness. *International journal of nursing sciences*, *3*(3), 242-251.

Theeke, L. A., Mallow, J. A., & Theeke, E. (2021). A Pilot One Group Feasibility, Acceptability, and Initial Efficacy Trial of LISTEN for Loneliness in Lonely Stroke Survivors. *SAGE open nursing*, *7*, 23779608211015154.

Theurer, K. A., Stone, R. I., Suto, M. J., Timonen, V., Brown, S. G., & Mortenson, W. B. (2021a). The Impact of Peer Mentoring on Loneliness, Depression, and Social Engagement in Long-Term Care. *Journal of applied gerontology : the official journal of the Southern Gerontological Society*, *40*(9), 1144-1152.

Theurer, K. A., Stone, R. I., Suto, M. J., Timonen, V., Brown, S. G., & Mortenson, W. B. (2021b). 'It makes life worthwhile!' Peer mentoring in long-term care-a feasibility study. *Aging & mental health*, *25*(10), 1887-1896.

Thimmapuram, J., Pargament, R., Bell, T., Schurk, H., & Madhusudhan, D. K. (2021). Heartfulness meditation improves loneliness and sleep in physicians and advance practice providers during COVID-19 pandemic. *Hospital practice (1995)*, *49*(3), 194-202.

Thomas, K. S., Akobundu, U., & Dosa, D. (2016). More Than A Meal? A Randomized Control Trial Comparing the Effects of Home-Delivered Meals Programs on Participants' Feelings of Loneliness. *The journals of gerontology. Series B, Psychological sciences and social sciences*, *71*(6), 1049-1058.

Tkatch, R., Wu, L., MacLeod, S., Ungar, R., Albright, L., Russell, D., . . . Yeh, C. S. (2021). Reducing loneliness and improving well-being among older adults with animatronic pets. *Aging & mental health*, *25*(7), 1239-1245.

Travers, C., & Bartlett, H. P. (2011). Silver Memories: implementation and evaluation of a unique radio program for older people. *Aging & mental health*, *15*(2), 169-177.

Vassilopoulos, S. P., Diakogiorgi, K., Brouzos, A., Moberly, N. J., & Chasioti, M. (2018). A problem-oriented group approach to reduce children's fears and concerns about the secondary school transition. *Journal of Psychologists and Counsellors in Schools*, *28*(1), 84-101.

Yarnoz, S., Plazaola, M., & Etxeberria, J. (2008). Adaptation to divorce: An attachment-based intervention with long-term divorced parents. *Journal of Divorce & Remarriage*, *49*(3), 291-307.
